# Risk prediction of mortality for patients with heart failure in England: observational study in primary care

**DOI:** 10.1002/ehf2.14250

**Published:** 2022-11-30

**Authors:** Alex Bottle, Roger Newson, Puji Faitna, Benedict Hayhoe, Martin R. Cowie

**Affiliations:** ^1^ School of Public Health Imperial College London London UK; ^2^ Comprehensive Cancer Centre King's College London London UK; ^3^ School of Cardiovascular Medicine & Sciences, Faculty of Life Sciences & Medicine King's College London London UK

**Keywords:** Heart failure, Risk prediction, Decision support, Electronic health records

## Abstract

**Aims:**

Many risk prediction models have been proposed for heart failure (HF), but few studies have used only information available to general practitioners (GPs) in primary care electronic health records (EHRs). We describe the predictors and performance of models built from GP‐based EHRs in two cohorts of patients 10 years apart.

**Methods and results:**

Linked primary and secondary care data for incident HF cases in England were extracted from the Clinical Practice Research Datalink for 2001–02 and 2011–12. Time‐to‐event models for all‐cause mortality were developed using a long list of potential baseline predictors. Discrimination and calibration were calculated. A total of 5966 patients in 156 general practices were diagnosed in 2001–02, and 12 827 patients in 331 practices were diagnosed in 2011–12. The 5‐year survival rate was 40.0% in 2001–02 and 40.2% in 2011–12, though the latter population were older, frailer, and more comorbid; for 2001–02, the 10‐year survival was 20.8% and 15‐year survival 11.1%. Consistent predictors included age, male sex, systolic blood pressure, body mass index, GP domiciliary visits before diagnosis, and some comorbidities. Model performance for both time windows was modest (*c* = 0.70), but calibration was generally excellent in both time periods.

**Conclusions:**

Information routinely available to UK GPs at the time of diagnosis of HF gives only modest predictive accuracy of all‐cause mortality, making it hard to decide on the type, place, and urgency of follow‐up. More consistent recording of data relevant to HF (such as echocardiography and natriuretic peptide results) in GP EHRs is needed to support accurate prediction of healthcare needs in individuals with HF.

## Introduction

Heart failure (HF) is a complex condition that affects around 900 000 in the United Kingdom and more than 26 million people worldwide.^1^ The prevalence is growing^2^; the UK incident and prevalent case numbers are rising,^3^ leading to increasing pressure on health services. One approach to offset the impact of HF on health resource use could include the use of a risk prediction model.^4^ HF is a highly heterogeneous condition in its presentation and prognosis; the majority of cases in England are now diagnosed via an emergency hospitalization, and a notable proportion of these admissions end in death.^5^ A better understanding of the risk of poor outcomes is likely to assist in identifying high‐risk patients early, providing opportunity for more timely effective management and better targeting of costly treatments.^6^, ^7^, ^8^ This could result in avoidance of healthcare costs as well as improved shared decision‐making between patients and clinicians.

Most HF patients in the United Kingdom are managed in primary care by general practitioners (GPs). However, limitations in access to investigations and specialist assessment, as well as overlap with other clinical conditions, mean that GPs face diagnostic uncertainty for patients whom they suspect have HF, which can contribute to difficulty choosing and initiating treatment.^9^, ^10^, ^11^ Many publications have proposed models for predicting death or other outcomes for different subgroups of HF patients, but significant variations in statistical methodologies and a lack of robust testing for biases have meant that no single risk model has been chosen.^7^, ^12^ A robust risk model should address both model discrimination and calibration, with calibration especially important as it reflects how well a model estimates absolute risk among patient groups. Risk models are based on population averages and can therefore be less accurate in estimating risk for some patient subgroups or outliers.^13^ As a result, it can be challenging for clinicians to appropriately identify who to use risk models for, as the consequences of applying risk estimates to an individual whose risk is not accurately reflected by the model could result in suboptimal treatment or even death.^13^, ^14^ Despite this, few publications on risk models report on model calibration or the handling of missing data.^6^, ^7^, ^12^

A recent systematic review of HF risk models for outcomes including death for patients in out‐of‐hospital settings found the majority of published models to have either a moderate or high risk of bias that could lead to misappropriate allocation of scarce resources.^12^ Di Tanna *et al*. applied the PROBAST tool to critically review each study's participant, predictor, outcome, and analysis selection.^12^, ^15^ Their commonest concerns were a risk of overfitting and no or limited validation. They called for more research into risk estimation in primary care and high‐risk or secondary prevention settings.

Most HF risk models use trial data on hospitalized patients, which may not reflect most HF patients, particularly those aged over 75 years.^16^, ^17^ Most ‘real‐world’ HF patients are elderly, have multiple comorbidities, and are managed in primary care,^16^ so utilizing real‐world datasets may provide more generalizable alternatives^18^; primary care datasets have the advantage of being representative of the large majority of patients managed in this setting. Additionally, younger HF patients could benefit from risk models for longer term survival beyond the commonly reported 1‐ or 5‐year range to empower clinicians and patients to make the required lifestyle decisions.^12^

With increased demand–supply mismatch for diagnostic testing, specialist input, and procedures in many healthcare systems after pandemic, assisting healthcare professionals with risk estimation tools could be important. This study aims to provide all‐cause mortality risk predictions for up to 10 years after diagnosis and its predictors in two UK community‐dwelling cohorts, 10 years apart, to better understand the extent to which electronic health records (EHRs) can guide GPs and HF patients in shared decision‐making and to further the debate about the value (or lack thereof) of such approaches. We note which predictors were consistent across models.

## Methods

### Data

The Clinical Practice Research Datalink (CPRD) is a database of pseudonymized electronic records from about 12% of UK general practices from 1987 to the present, considered broadly representative of the UK population.^19^ Primary care records are linked nationally for English practices to hospital admissions [Hospital Episode Statistics (HES)] and the death registry (Office for National Statistics).

### Cohort definition

A proxy HF diagnosis date was defined as the earliest mention of an HF clinical code (see Supporting Information, *Appendix* *S1*) in either primary or secondary care data. HF diagnosis dates could be in calendar years 2001–02 (‘Cohort 1’) or in calendar years 2011–12 (‘Cohort 2’). We defined ‘diagnosis setting’ as whether the first HF record was in primary or secondary care. As per our previous work with CPRD,^5^ we ran sensitivity analyses using the first date of GP prescription of a loop diuretic as the HF diagnosis date, recognizing that some GPs will treat the symptoms of patients with suspected HF before or while formally investigating for HF.

### Predictors

A long list of potential predictors was derived by literature review, taking into account what was recorded with at least 1% prevalence in CPRD. Predictors included in all models compared in the test set were as described below. Gender and baseline age were modelled as a pair of cubic splines for the two genders, with reference points at ages 50, 60, 70, 80, and 90. Other predictors were as follows: body mass index (BMI) (kg/m^2^), systolic blood pressure (BP) (mmHg), and electronic frailty index^20^ based on polypharmacy in the previous 1 year and other deficits over the previous 5 years (modelled as cubic splines interactive with age); Index of Multiple Deprivation (IMD, an area‐level socio‐economic status measure) 2010 twentile (modelled as an additive cubic spline); cigarette smoking category (lifelong non‐smoker, ex‐smoker, <10 cigarettes/day, 10–19 cigarettes/day, or 20+ cigarettes/day); diabetes status (none, Type I, or Type II); ethnicity from HES (White, non‐White, or unknown); and a list of binary predictors. The binary predictors were defined using CPRD records in the previous 5 years, CPRD records in the previous 1 year, HES records in the previous 1 year, or combined CPRD and HES records in the previous 5 years. The CPRD binary predictors measured over the previous 5 years were as follows: comorbidities (atrial fibrillation, arrhythmia other than atrial fibrillation, hypertension, renal diseases, myocarditis, acute myocardial infarction, congenital heart disease, coronary heart disease, chronic pulmonary disease, stroke, and peripheral vascular disease); widowed or bereaved; and recorded HF symptom presence (breathlessness/shortness of breath/shortness of breath on exertion, fatigue, and ankle swelling). The CPRD binary predictors measured over the previous year were as follows: appointment‐type presence (GP appointment of 4+ min, practice nurse appointment of 4+ min, home visit appointment, out‐of‐hours GP appointment, GP reported non‐attendance, and practice nurse reported non‐attendance); CPRD‐recorded accident and emergency department visit; CPRD‐recorded outpatient department appointment; and CPRD‐recorded prescriptions for a list of classes of drugs. These classes of drugs were as follows: beta‐blockers [British National Formulary (BNF) Chapter 2.4]; thiazide‐related diuretics (BNF Chapter 2.2.1); loop diuretics (BNF Chapter 2.2.2); aldosterone antagonists (spironolactone or eplerenone); renin–angiotensin system (RAS) drugs (BNF Chapter 2.5.5); glucocorticoid therapy (BNF Chapter 6.3.2); and atypical antipsychotics (BNF Chapter 4.2.1.2 and/or any of 10 individual drug names). The HES binary predictors (presence indicators over the previous 1 year) were as follows: three procedures (coronary artery bypass surgery, percutaneous transluminal coronary angioplasty, and pacemaker); any hospital dialysis; elective bed admission without HF primary diagnosis; emergency non‐HF bed admission (1 day only); emergency non‐HF bed admission (at least one night); and any hospital admissions with primary diagnosis in the US Agency for Healthcare Research and Quality's Clinical Classification Software (CCS) Categories 086 (cataract), 122 (pneumonia except that caused by TB or STD), 127 [chronic obstructive pulmonary disease (COPD) and bronchiectasis], and 134 (other upper respiratory disease). The CCS system was devised by the Agency for Healthcare Research and Quality as a general‐purpose way of grouping ICD10 codes into homogeneous groups. The binary indicator derived from combined CPRD and HES data in the previous 5 years was living alone.

### Statistical analysis

Each cohort was split randomly and equally into training and test sets, each with 174 practices. We fitted models in the training set and compared their performance (in terms of discrimination and calibration) in the test set. A series of survival regression models were fitted to the data in the training set and used to predict *k*‐year survival probabilities for *k* from 1 to 10 from a list of baseline covariates available at diagnosis time. Models were Gompertz (with intercept parameters equal to baseline hazard rates, effect parameters equal to hazard ratios, and a per‐year exponential hazard rate trend parameter), Weibull (with intercept parameters equal to Year 1 death rates, effect parameters equal to hazard ratios, and a time power hazard rate trend parameter), or Cox (with no intercept and an arbitrary baseline hazard). We used cubic reference splines for continuous covariates, defined using the ‘polyspline’ Stata add‐on package, which implements the methods of Newson,^21^ and level indicators for binary and other categorical factors. Imputation of missing values for continuous variables was done using a cubic spline model in gender and age to impute missing BMIs and systolic BPs, and imputing missing IMD twentiles to twentile 10. This practice is similar to that used by QRISK^22^ and is a lot less computer intensive than multiple imputation.

For model discrimination, Harrell's *c* indices of Year 1 survival probability with respect to survival were estimated using the add‐on ‘somersd’ package (Newson^23^) to compute confidence intervals (CIs) and *P* values clustered by general practice. Calibration was measured using decile plots for *k*‐year survival probability for *k* from 1 to 10. For each model, we divided the whole cohort, and the sub‐cohorts for the sub‐models, into deciles of *k*‐year survival probability. For each decile, we defined predicted *k*‐year survival probability as the mean *k*‐year survival probability for that model for that decile and estimated observed *k*‐year survival probability using the Kaplan–Meier curve for that decile, with normal‐theory bootstrap CIs, using the conventional normalizing complementary log–log transform and clustered by general practice. The predicted *k*‐year survival probabilities, and the observed *k*‐year survival probabilities with confidence limits, were plotted against risk decile to form a decile plot. Stata Version 16 was used throughout.

### Ethics

We have approval from the Secretary of State and the Health Research Authority under Regulation 5 of the Health Service (Control of Patient Information) Regulations 2002 to hold confidential data and analyse them for research purposes [Confidentiality Advisory Group (CAG) Ref 15/CAG/0005]. We have approval to use them for research and measuring quality of delivery of healthcare from the London—South East Research Ethics Committee (Ref 20/LO/0611). Access to CPRD for this study was approved by their Independent Scientific Advisory Committee, Protocol Number 18_109.

## Results

Heart failure diagnostic codes were identified for 5966 patients in 156 practices in Cohort 1, and 12 827 patients in 331 practices were diagnosed in Cohort 2. Diagnoses could be reported first by CPRD in a primary care setting (6964 patients in 342 practices) or reported by HES in a hospital setting (11 829 patients in 347 practices).

*Tables* *1A*, *1B*, *1C* show that, compared with Cohort 1 patients, Cohort 2 patients had a greater proportion aged 85+, lower BP and cholesterol, and greater comorbidity (especially atrial fibrillation and diabetes; the estimated glomerular filtration rate and renal disease were poorly recorded in Cohort 1) and more likely to live alone. They also had greater use of beta‐blockers and RAS medications.

**Table 1A:** Quantitative patient characteristics at HF diagnosis in each cohort

Quantitative variable	Cohort 1			Cohort 2		
	*N* present	Mean	SD	*N* present	Mean	SD
Age in diagnosis year	5981	76.9	11.0	12 830	77.5	12.1
Electronic frailty index (eFI)	5981	0.18	0.1	12 830	0.22	0.1
BMI (kg/m^2^)	2914	27.5	5.7	10 219	28.1	6.5
Systolic blood pressure (mmHg)	5262	145.4	22.8	12 590	133.6	19.3
Diastolic blood pressure (mmHg)	5262	80.0	11.7	12 590	74.8	11.4
Serum creatinine (μmol/L)	3480	108.3	43.0	12 284	101.9	50.9
Haemoglobin (g/dL)	3151	13.1	1.9	11 903	12.8	1.9

BMI, body mass index; HF, heart failure.

**Table 1B:** Categorical patient characteristics at HF diagnosis in each cohort

Factor level	Cohort 1		Cohort 2	
	*N*	%	*N*	%
All patients				
Total	5981	100	12 830	100
Patient gender				
Male	2959	49.5	6623	51.6
Female	3022	50.5	6207	48.4
Age group in diagnosis year				
<45	57	1.0	162	1.3
45–64	716	12.0	1684	13.1
65–74	1324	22.1	2487	19.4
75–84	2383	39.8	4357	34.0
85+	1501	25.1	4140	32.3
IMD 2010 quintile				
1	967	16.2	2471	19.3
2	1399	23.4	3026	23.6
3	1252	20.9	2711	21.1
4	1214	20.3	2513	19.6
5	1130	18.9	2101	16.4
Unknown	19	0.3	8	0.1
HES ethnicity				
White	5037	84.2	12 251	95.5
Non‐White	105	1.8	392	3.1
Unknown	839	14.0	187	1.5
Source of first HF diagnosis				
GP consultation (CPRD)	3027	50.6	3955	30.8
Hospital admission (HES)	2954	49.4	8875	69.2
Number of comorbidities (group)				
0	904	15.1	677	5.3
1	1689	28.2	1822	14.2
2	1560	26.1	2929	22.8
3	1046	17.5	2965	23.1
4+	782	13.1	4437	34.6
Electronic frailty index (eFI) category				
Fit	1597	26.7	1755	13.7
Mild frailty	3109	52.0	5837	45.5
Moderate frailty	1127	18.8	4296	33.5
Severe frailty	148	2.5	942	7.3
Smoking category				
Non‐smoker	626	10.5	5073	39.5
Ex‐smoker	422	7.1	4830	37.6
Current smoker	1893	31.7	2105	16.4
Unknown	3040	50.8	822	6.4
Alcohol drinking group				
Non‐drinker	232	3.9	2445	19.1
Light, moderate, or unspecified	1675	28.0	4703	36.7
Heavy or alcoholic	237	4.0	730	5.7
Unknown	3837	64.2	4952	38.6
BMI group				
Underweight	95	1.6	354	2.8
Normal	910	15.2	3137	24.5
Overweight	1100	18.4	3440	26.8
Obese	836	14.0	3334	26.0
Unknown	3040	50.8	2565	20.0
Diabetes status				
No diabetes	4999	83.6	9805	76.4
Type 1 diabetes	378	6.3	732	5.7
Type 2 diabetes	604	10.1	2293	17.9

BMI, body mass index; CPRD, Clinical Practice Research Datalink; GP, general practitioner; HES, Hospital Episode Statistics; HF, heart failure; IMD, Index of Multiple Deprivation.

**Table 1C:** Binary patient characteristics at HF diagnosis in each cohort

Factor	*N*	%	*N*	%
HF symptoms up to diagnosis				
Presence of: any heart failure symptom	2790	46.6	7173	55.9
Presence of: breathlessness/SOB/SOBE	2161	36.1	5809	45.3
Presence of: fatigue	629	10.5	1685	13.1
Presence of: ankle swelling	584	9.8	1716	13.4
First‐symptom presence of: breathlessness/SOB/SOBE	1860	31.1	4934	38.5
First‐symptom presence of: fatigue	498	8.3	1200	9.4
First‐symptom presence of: ankle swelling	440	7.4	1127	8.8
Physiological indicators				
eGFR below 60 mL/min^a^	23	41.1	3469	43.3
Social vulnerability indicators				
Living alone	342	5.7	1219	9.5
Widowed or bereaved	376	6.3	761	5.9
Comorbidity components				
Comorbidity: 1 Atrial fibrillation	1533	25.6	5397	42.1
Comorbidity: 2 Arrhythmia other than atrial fibrillation	617	10.3	2346	18.3
Comorbidity: 3 Diabetes	979	16.4	3023	23.6
Comorbidity: 4 Hypertension	2528	42.3	8966	69.9
Comorbidity: 5 Renal diseases	363	6.1	4069	31.7
Comorbidity: 6 Myocarditis	82	1.4	329	2.6
Comorbidity: 7 Acute myocardial infarction	949	15.9	2229	17.4
Comorbidity: 8 Congenital heart disease	20	0.3	97	0.8
Comorbidity: 9 Coronary heart disease	2057	34.4	5119	39.9
Comorbidity: 10 Chronic pulmonary disease	1281	21.4	3440	26.8
Comorbidity: 11 Stroke	458	7.7	1140	8.9
Comorbidity: 12 Peripheral vascular disease	517	8.6	1482	11.6
Baseline NHS contacts in previous year				
CABG	37	0.6	92	0.7
PTCA	33	0.6	314	2.4
Pacemaker	52	0.9	205	1.6
Any hospital bed admission	2682	44.8	7311	57.0
Elective bed admission without HF primary diagnosis	1314	22.0	3567	27.8
Emergency bed admission without HF primary diagnosis	1940	32.4	5593	43.6
Emergency non‐HF bed admission (1 day only)	137	2.3	1045	8.1
Emergency non‐HF bed admission (at least one night)	1873	31.3	5173	40.3
4+ min GP appointment	4700	78.6	12 251	95.5
4+ min practice nurse appointment	2614	43.7	9243	72.0
Home visit appointment	2108	35.2	4317	33.6
Out‐of‐hours appointment	862	14.4	2378	18.5
CPRD‐recorded A&E visit	348	5.8	3970	30.9
CPRD‐recorded OPD appointment	1504	25.1	8228	64.1
Beta‐blockers (BNF Chapter 2.4)	1453	24.3	5013	39.1
Thiazide‐related diuretics (BNF Chapter 2.2.1)	1158	19.4	2434	19.0
Loop diuretics (BNF Chapter 2.2.2)	2349	39.3	5504	42.9
Aldosterone antagonists (spironolactone or eplerenone)	186	3.1	709	5.5
Renin–angiotensin system (RAS) drugs (BNF Chapter 2.5.5)	1903	31.8	6967	54.3
Glucocorticoid therapy (BNF Chapter 6.3.2)	870	14.5	2464	19.2
Atypical antipsychotics (BNF Chapter 4.2.1.2 or drug names)	59	1.0	146	1.1
Primary CCS groups for HES admissions in previous year				
086 Cataract	176	2.9	410	3.2
122 Pneumonia (except that caused by tuberculosis or sexually transmitted diseases	70	1.2	474	3.7
127 Chronic obstructive pulmonary disease and bronchiectasis	122	2.0	342	2.7
134 Other upper respiratory disease	89	1.5	345	2.7

A&E, accident and emergency department; BNF, British National Formulary; CABG, coronary artery bypass graft; CCS, Clinical Classification Software; CPRD, Clinical Practice Research Datalink; eGFR, estimated glomerular filtration rate; GP, general practitioner; HES, Hospital Episode Statistics; HF, heart failure; NHS, National Health Service; OPD, outpatient department; PTCA, percutaneous transluminal coronary angioplasty; SOB, shortness of breath; SOBE, shortness of breath on exertion.

^a^ Only recorded for 56 patients in Cohort 1 and 8005 patients in Cohort 2.

The 5‐year survival rate was 40.0% (95% CI, 38.3–41.7%) in Cohort 1 and 40.2% (95% CI, 39.1–41.4%) in Cohort 2. In Cohort 1, 10‐year survival was 20.8% (95% CI, 19.3–22.3%), and 15‐year survival was 11.1% (95% CI, 10.1–12.3%).

Standard error inspection and the usual tests did not show problems with multicollinearity. Model performance in the test set for each cohort was at best fair (*c* = 0.70 overall, with some variation by cohort and diagnosis setting: *Table* *2*). We also examined whether discrimination was different for shorter follow‐up lengths after diagnosis (i.e., within 3, 6, and 12 months of diagnosis) than for 60 months, and it was not (differences in *c* statistics were <0.01; data not shown).

**Table 2:** Discrimination (*c* statistic) for each Cox model and diagnosis setting

Model	Training set	Test set
Cohort 1, HF recorded first in primary care	0.72	0.69
Cohort 1, HF recorded first in hospital data	0.70	0.65
Cohort 2, HF recorded first in primary care	0.75	0.71
Cohort 2, HF recorded first in hospital data	0.69	0.66
All patients (both cohorts, both diagnosis settings combined)	0.71	0.70

HF, heart failure.

Cohort 1: diagnosis in 2001–02; Cohort 2: diagnosis in 2011–12.

**Figure 1:**
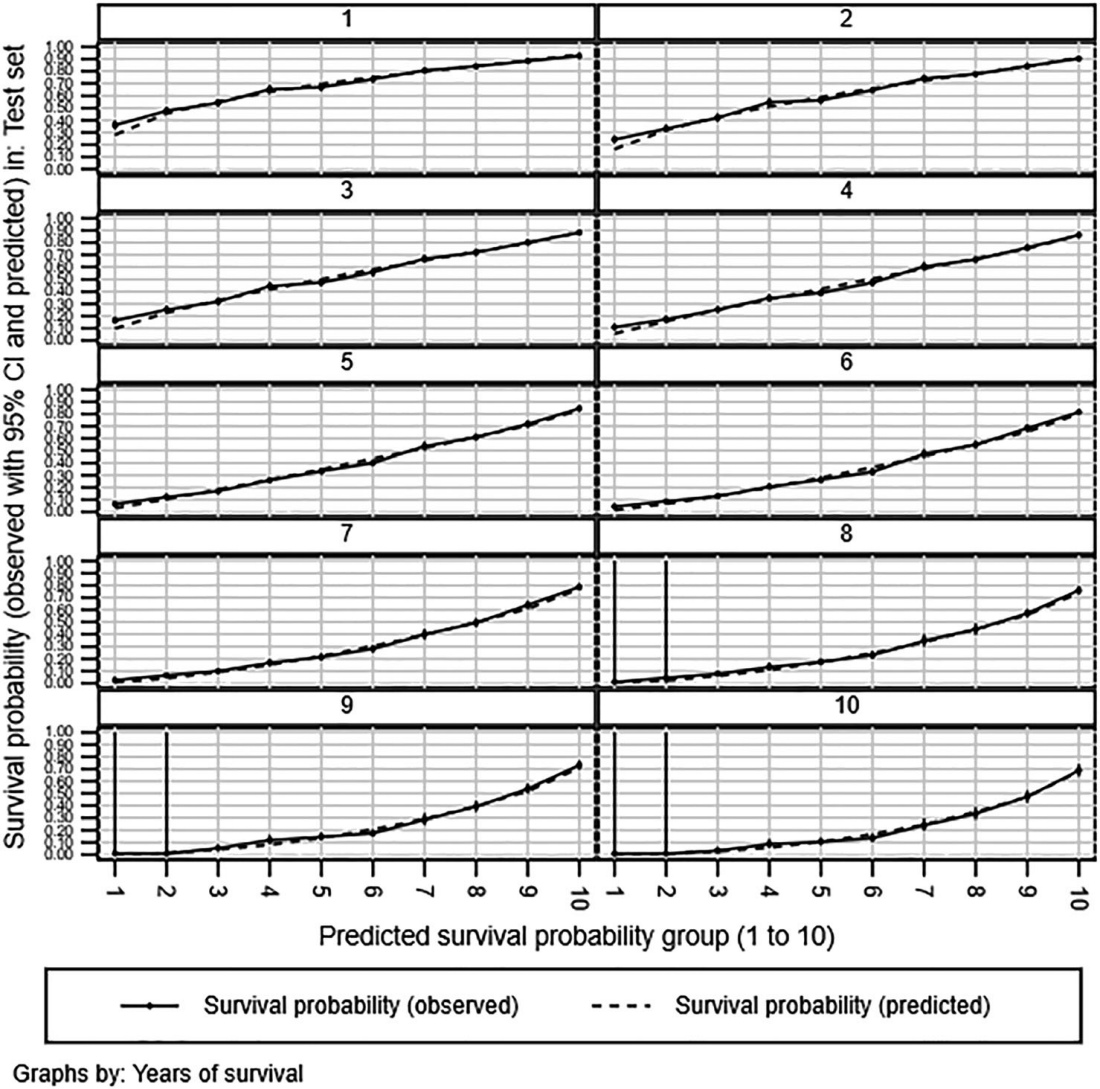
Calibration curves for Cox model by decile (‘group’) of predicted mortality risk. CI, confidence interval.

Calibration was good. Fitting four separate sets of hazard ratios, one per combination of cohort and diagnosis setting, reduced model performance; calibration was superior for the Cox over the Weibull and Gompertz models in the test set. *Figure* *1* gives the overall test set calibration for the final Cox model; Supporting Information, *Appendix* *S1* gives the same plots for the Weibull and Gompertz. Calibration was very similar for each cohort and diagnosis setting (not shown). The model showed some overestimation in low‐risk patients (risk deciles 1–4) in the first year since diagnosis but otherwise fitted well.

The full sets of hazard ratios for all‐cause mortality since HF diagnosis, split by cohort and diagnosis setting, are given in Supporting Information, *Appendix* *S1*. Given the large size of these four tables, we have summarized the statistical significance and effect sizes of the predictors in *Table* *3*, with hazard ratios of <0.80 or >1.20 and *P* < 0.05 marked as ‘YY’. Hazard ratios closer to 1 but with *P* < 0.05 are marked as ‘Y’.

**Table 3:** Summary of direction of any association with mortality, statistical significance, and effect size of each predictor following HF diagnosis, stratified by cohort and diagnosis setting (where HF was first recorded)

Predictor	Primary care diagnosed, Cohort 1	Primary care diagnosed, Cohort 2	Hospital diagnosed, Cohort 1	Hospital diagnosed, Cohort 2
Age	++	++	++	++
Sex (lower mortality for older women compared with older men)	++	++	++	++
BMI (higher risk for BMI under 25 in older people)	++	++	++	++
Systolic BP		++	++	+
Frailty (eFI)		++	++	−
Deprivation (IMD)			++	
Smoking	++ (heavy)	++ (mod, heavy)	++ (heavy)	++ (mod, heavy)
Diabetes	++ (types 1, 2)		++ (type 2)	++ (types 1, 2)
Ethnicity (White, non‐White)	++ (unknown)	++ (unknown)	++ (unknown)	++ (unknown; non‐White lower risk)
Atrial fibrillation				
Other arrhythmias			−	−
Hypertension				
Renal disease	++		++	++
Myocarditis				
Prior acute myocardial infarction				
Congenital heart disease				
Coronary heart disease				−
COPD		++	++	++
Stroke				
Peripheral vascular disease	++	++	++	
Haemoglobin				
Serum creatinine				
Living alone				
Widowed or bereaved				
HF symptoms		+− (fatigue = lower risk, ankle swelling = higher)		
Coronary artery bypass graft				−
Percutaneous coronary intervention	−	−		−
Elective non‐HF admission	+			
Emergency non‐HF same‐day admission			++	
Emergency non‐HF admission	++			++
GP appointments				
Nurse appointments				−
Home visit	++	++	++	++
Out‐of‐hours appointment				
GP appointment non‐attendance				
Nurse appointment non‐attendance				
Emergency department visit				
Outpatient department appointment				
Beta‐blocker				−
Thiazide‐related diuretics				
Loop diuretics				
Aldosterone antagonists		++		++
Renin–angiotensin system drugs				−
Glucocorticoid therapy				
Atypical antipsychotics	++			
CCS group: 086 Cataract				
CCS group: 122 Pneumonia (except that caused by TB or STD)				
CCS group: 127 COPD and bronchiectasis		++		
CCS group: 134 Other upper respiratory disease				

BMI, body mass index; BP, blood pressure; CCS, Clinical Classification Software: these variables refer to pre‐diagnosis admissions for the specified diagnoses; COPD, chronic obstructive pulmonary disease; eFI, electronic frailty index; GP, general practitioner; HF, heart failure; HR, hazard ratio; IMD, Index of Multiple Deprivation; STD, sexually transmitted disease; TB, tuberculosis.

Key: + = *P* < 0.05 but 0.80 < HR < 1.20; ++ = *P* < 0.05 and (HR ≤ 0.80 or HR ≥ 1.20); − = *P* < 0.05 but 0.80 < HR < 1.00; −− = *P* < 0.05 and HR ≤ 0.80; else blank.

## Discussion

### Summary of main findings

In our analysis of primary care EHRs linked to administrative hospital data, we found that the best fitting model was Cox with baseline hazards stratified by cohort and diagnosis setting. Model discrimination was modest, with a *c* statistic of around 0.70. Despite some overprediction in low‐risk patients in the year after diagnosis, calibration was very good. The 5‐year survival was 40% in both cohorts overall. Mortality predictors were largely unchanged over the 10 years separating the two cohorts, with factors such as older age, male gender, higher BP, BMI (higher risk for BMI under 25 in older people), home visits before diagnosis, smoking, and some comorbidities (renal disease, COPD, and peripheral vascular disease) consistent across cohorts and diagnosis settings.

### Comparison with previous studies

A 2020 systematic review^12^ found 58 risk prediction models for HF patients, with various outcomes: ‘the discriminatory ability for predicting all‐cause mortality, cardiovascular death, and composite endpoints was generally better than for HF hospitalization. 105 distinct predictor variables were identified. Predictors included in >5 publications were: N‐terminal prohormone brain‐natriuretic peptide, creatinine, blood urea nitrogen, systolic blood pressure, sodium, NYHA class, left ventricular ejection fraction, heart rate, and characteristics including male sex, diabetes, age, and BMI’. Our data had the last four of these and systolic BP, and we also found them to be consistent predictors. In the all‐cause mortality models, discrimination ranged from 0.66 to 0.84, but in the five studies on non‐randomized controlled trial chronic HF patients in the community and therefore relevant to our study, the range was 0.68–0.74, comparable to our models.

The inverse association between use of mineralocorticoid receptor antagonist and outcome has been reported before on several occasions in other real‐world datasets.^24^, ^25^ It is presumably because the use of such agents (much lower than almost universal use of at least some dose of RAS inhibitor or beta‐blocker) identifies a particularly high‐risk subgroup of patients with substantial comorbidity.

### Strengths and limitations

Clinical Practice Research Datalink is broadly representative of the general UK community‐dwelling population and gives a broad representation of the data GPs have available to them in making decisions. Its linkage to HES and the death registry means national coverage for those outcomes. CPRD data are entered by GPs during routine consultations and not for the purpose of research, and it is recognized that coding quality is variable.^26^ However, in a validation study with CPRD in 2001, questionnaires concerning 1200 patients flagged as having HF were sent to GPs to confirm the diagnosis. Of the 1146 returned, in only 72 patients did the GP not confirm the diagnosis. A further 136 reported a history of HF that was not recorded in the computer file.^27^ Other CPRD limitations include missing values, which we imputed using splines rather than the more computer‐intensive multiple imputation. This was a pragmatic choice given the size of the dataset and number of predictors and is similar to the QRISK algorithm approach.^22^ Most patients had no BNP or HF type recorded; only 19/58 models in the 2020 systematic review reported the HF subtype.^12^ The ejection fraction and, for Cohort 2, BNP values were only recorded in a small minority of patients. Ejection fractions are determined via echocardiography in hospital and, to be recorded in primary care data, need to be sent to the practice and entered and coded correctly by practice staff. It is not clear why, even in those patients who did receive a BNP test in Cohort 2, few had their results in the primary care records in the same way as other blood test results. It seems reasonable to conclude that, although these data may be available to GPs within the EHR, in the body of hospital letters appended to EHRs as attachments, for example, they are likely to be less than immediately visible and so are inaccessible to automated risk prediction tools or decision support systems.

We used the first recorded mention of HF as the date of diagnosis. For some GPs, HF may be a working diagnosis, which they may or may not record formally, although they carry out confirmatory investigations. Consequently, we undertook a sensitivity analysis using the date of first loop diuretic prescription to indicate the date when the GP first suspected HF that was only confirmed later. This did not change the findings meaningfully.

Risk assessment tools need to be updated over time to account for therapy advances, but to study longer term prognosis requires a significant time lag, as here. This inevitably means that covariates measured at baseline—when the GP made the initial management decisions—reflect clinical practice at that time, which in our study was 2001–02 and 2011–12. Despite this, it is notable that overall 5‐year mortality and which predictors were significant changed little during that time. It should be remembered that the associations between predictors and the outcome that were found in our analysis are not necessarily causal.

Our aim was to fit models with information available to clinicians at the point of diagnosis. This therefore precluded the use of time‐varying covariates to capture changes after diagnosis. For continuous variables with multiple measures, we took the measure just before diagnosis, but other options are available that aim to capture the variation or trends in these variables. These include two‐stage approaches involving a linear mixed model and then a survival model (the predicted BP trajectory is plugged into the survival model), and a joint longitudinal and survival model. A review found six approaches but without consensus on which to use^28^; machine learning was one and could also have been applied to our data and modelling framework instead of Cox regression, for instance, using random forest survival analysis. There has been a lot of discussion around machine learning for prediction with EHRs, with the potential for superior prediction.^29^

We have reported mortality, but of course poor outcomes aside from death may affect patients with HF, and, like everyone else, they also value the ability to work, to travel, and to socialize. CPRD lacks this information; we report elsewhere the risk of emergency hospitalization.^30^

## Conclusions

Electronic health records in primary care can accommodate sophisticated risk prediction algorithms, which are likely to be of significant value in supporting clinicians in identification of high‐risk individuals among their case load, offering timely and appropriately targeted management, and providing detailed risk information to patients to support evidence‐based shared decisions about care. However, although our models for predicting all‐cause mortality calibrate well, with some consistent predictors across our two cohorts, they have modest discrimination using only the information available to GPs at the time of HF diagnosis and initial decision‐making. This makes it hard to use these models to support decisions on the type, place, and urgency of management and follow‐up. This work highlights the need for better recording of key metrics such as ejection fraction and BNP levels in GP EHRs; consistent coding of these data will support the development of effective prediction models likely to be of significant value in identification of at‐risk patients and informing conversations with patients and their families around prognosis, care, and treatment options.

## Conflict of interest

B.H. is a GP working in NHS primary care and clinical lead for research and development for eConsult Health Ltd (a provider of electronic consultations in NHS primary, secondary, and urgent/emergency care). There are no relevant conflicts for the other authors.

## Funding

This work was supported by the British Heart Foundation (PG/18/3/33515).

## Supporting information


**Appendix S1.** Supporting Information.
