# Supplementary material for: Risk prediction of mortality for patients with heart failure in England: observational study in primary care
Source: ESC Heart Fail. 2022 Nov 30;10(2):824–33. doi: 10.1002/ehf2.14250 (PMC10053260; doi:10.1002/ehf2.14250)

## Appendices

**Further details of the survival analysis modelling**

There were three stages of modelling. The first stage compared unstratified Gompertz, Weibull and Cox models regarding estimation of k-year survival probabilities for k from 1 to 10 (which in an unstratified model are monotonically increasing in each other). The second stage compared only Cox kitchen-sink models (stratified by diagnosis time window and diagnosis setting), because the Cox model calibrated a lot better than the Gompertz and Weibull models, and focussed on 3-month, 6-month, 12-month and 60-month survival times. The third stage compared simplified Cox models (simplified using backwards stepwise regression n the training set), and again focussed on 3-month, 6-moonth, 12-month and 60-month survival probabilities. All 3 stages contained models with a single vector of hazard ratios and also tetrads of models with a separate vector of hazard ratio for each of the 4 combinations of diagnosis window and diagnosis setting. And, for Window 2, variant models with the extra A&E and OPD predictors were also fitted in all 3 stages.

Table A1. Further patient characteristics.

|  | *Window 1* |  |  | *Window 2* |  |  | *Total* |  |  |
| --- | --- | --- | --- | --- | --- | --- | --- | --- | --- |
| *Factor* | *N present* | *Frequency* | *Percent* | *N present* | *Frequency* | *Percent* | *N present* | *Frequency* | *Percent* |
| **HF symptoms up to diagnosis:** |  |  |  |  |  |  |  |  |  |
| Presence of: Any heart failure symptom | 5981 | 2790 | 46.6 | 12830 | 7173 | 55.9 | 18811 | 9963 | 53.0 |
| Presence of: Breathlessness/SOB/SOBE | 5981 | 2161 | 36.1 | 12830 | 5809 | 45.3 | 18811 | 7970 | 42.4 |
| Presence of: Fatigue | 5981 | 629 | 10.5 | 12830 | 1685 | 13.1 | 18811 | 2314 | 12.3 |
| Presence of: Ankle swelling | 5981 | 584 | 9.8 | 12830 | 1716 | 13.4 | 18811 | 2300 | 12.2 |
| First-symptom presence of: Breathlessness/SOB/SOBE | 5981 | 1860 | 31.1 | 12830 | 4934 | 38.5 | 18811 | 6794 | 36.1 |
| First-symptom presence of: Fatigue | 5981 | 498 | 8.3 | 12830 | 1200 | 9.4 | 18811 | 1698 | 9.0 |
| First-symptom presence of: Ankle swelling | 5981 | 440 | 7.4 | 12830 | 1127 | 8.8 | 18811 | 1567 | 8.3 |
| **Electronic frailty index (eFI) deficits:** |  |  |  |  |  |  |  |  |  |
| Presence of deficit: 1 Activity limitation | 5981 | 118 | 2.0 | 12830 | 152 | 1.2 | 18811 | 270 | 1.4 |
| Presence of deficit: 2 Anaemia & haematinic deficiency | 5981 | 2480 | 41.5 | 12830 | 9628 | 75.0 | 18811 | 12108 | 64.4 |
| Presence of deficit: 3 Arthritis | 5981 | 1393 | 23.3 | 12830 | 2359 | 18.4 | 18811 | 3752 | 19.9 |
| Presence of deficit: 4 Atrial fibrillation | 5981 | 843 | 14.1 | 12830 | 3498 | 27.3 | 18811 | 4341 | 23.1 |
| Presence of deficit: 5 Cerebrovascular disease | 5981 | 615 | 10.3 | 12830 | 1546 | 12.0 | 18811 | 2161 | 11.5 |
| Presence of deficit: 6 Chronic kidney disease | 5981 | 142 | 2.4 | 12830 | 6507 | 50.7 | 18811 | 6649 | 35.3 |
| Presence of deficit: 7 Diabetes | 5981 | 895 | 15.0 | 12830 | 2918 | 22.7 | 18811 | 3813 | 20.3 |
| Presence of deficit: 8 Dizziness | 5981 | 1043 | 17.4 | 12830 | 2421 | 18.9 | 18811 | 3464 | 18.4 |
| Presence of deficit: 9 Dyspnoea | 5981 | 2356 | 39.4 | 12830 | 5474 | 42.7 | 18811 | 7830 | 41.6 |
| Presence of deficit: 10 Falls | 5981 | 922 | 15.4 | 12830 | 2513 | 19.6 | 18811 | 3435 | 18.3 |
| Presence of deficit: 11 Foot problems | 5981 | 66 | 1.1 | 12830 | 856 | 6.7 | 18811 | 922 | 4.9 |
| Presence of deficit: 12 Fragility fracture | 5981 | 269 | 4.5 | 12830 | 822 | 6.4 | 18811 | 1091 | 5.8 |
| Presence of deficit: 13 Hearing impairment | 5981 | 563 | 9.4 | 12830 | 1706 | 13.3 | 18811 | 2269 | 12.1 |
| Presence of deficit: 14 Heart failure | 5981 | 3315 | 55.4 | 12830 | 4398 | 34.3 | 18811 | 7713 | 41.0 |
| Presence of deficit: 15 Heart valve disease | 5981 | 89 | 1.5 | 12830 | 548 | 4.3 | 18811 | 637 | 3.4 |
| Presence of deficit: 16 Housebound | 5981 | 1472 | 24.6 | 12830 | 5715 | 44.5 | 18811 | 7187 | 38.2 |
| Presence of deficit: 17 Hypertension | 5981 | 4863 | 81.3 | 12830 | 11806 | 92.0 | 18811 | 16669 | 88.6 |
| Presence of deficit: 18 Hypotension / syncope | 5981 | 648 | 10.8 | 12830 | 1487 | 11.6 | 18811 | 2135 | 11.3 |
| Presence of deficit: 19 Ischaemic heart disease | 5981 | 2450 | 41.0 | 12830 | 4387 | 34.2 | 18811 | 6837 | 36.3 |
| Presence of deficit: 20 Memory & cognitive problems | 5981 | 321 | 5.4 | 12830 | 1097 | 8.6 | 18811 | 1418 | 7.5 |
| Presence of deficit: 21 Mobility and transfer problems | 5981 | 321 | 5.4 | 12830 | 827 | 6.4 | 18811 | 1148 | 6.1 |
| Presence of deficit: 22 Osteoporosis | 5981 | 227 | 3.8 | 12830 | 891 | 6.9 | 18811 | 1118 | 5.9 |
| Presence of deficit: 23 Parkinsonism & tremor | 5981 | 117 | 2.0 | 12830 | 200 | 1.6 | 18811 | 317 | 1.7 |
| Presence of deficit: 24 Peptic ulcer | 5981 | 137 | 2.3 | 12830 | 253 | 2.0 | 18811 | 390 | 2.1 |
| Presence of deficit: 25 Peripheral vascular disease | 5981 | 184 | 3.1 | 12830 | 787 | 6.1 | 18811 | 971 | 5.2 |
| Presence of deficit: 26 Polypharmacy | 5981 | 5144 | 86.0 | 12830 | 11626 | 90.6 | 18811 | 16770 | 89.1 |
| Presence of deficit: 27 Requirement for care | 5981 | 56 | 0.9 | 12830 | 315 | 2.5 | 18811 | 371 | 2.0 |
| Presence of deficit: 28 Respiratory disease | 5981 | 1430 | 23.9 | 12830 | 3555 | 27.7 | 18811 | 4985 | 26.5 |
| Presence of deficit: 29 Skin ulcer | 5981 | 676 | 11.3 | 12830 | 1336 | 10.4 | 18811 | 2012 | 10.7 |
| Presence of deficit: 30 Sleep disturbance | 5981 | 896 | 15.0 | 12830 | 1022 | 8.0 | 18811 | 1918 | 10.2 |
| Presence of deficit: 31 Social vulnerability | 5981 | 174 | 2.9 | 12830 | 436 | 3.4 | 18811 | 610 | 3.2 |
| Presence of deficit: 32 Thyroid disease | 5981 | 573 | 9.6 | 12830 | 2414 | 18.8 | 18811 | 2987 | 15.9 |
| Presence of deficit: 33 Urinary incontinence | 5981 | 268 | 4.5 | 12830 | 712 | 5.5 | 18811 | 980 | 5.2 |
| Presence of deficit: 34 Urinary system disease | 5981 | 1536 | 25.7 | 12830 | 3637 | 28.3 | 18811 | 5173 | 27.5 |
| Presence of deficit: 35 Visual impairment | 5981 | 959 | 16.0 | 12830 | 2621 | 20.4 | 18811 | 3580 | 19.0 |
| Presence of deficit: 36 Weight loss & anorexia | 5981 | 286 | 4.8 | 12830 | 762 | 5.9 | 18811 | 1048 | 5.6 |
| **Baseline NHS contacts in previous year:** |  |  |  |  |  |  |  |  |  |
| CABG | 5981 | 37 | 0.6 | 12830 | 92 | 0.7 | 18811 | 129 | 0.7 |
| PTCA | 5981 | 33 | 0.6 | 12830 | 314 | 2.4 | 18811 | 347 | 1.8 |
| Pacemaker | 5981 | 52 | 0.9 | 12830 | 205 | 1.6 | 18811 | 257 | 1.4 |
| ICD | 5981 | 0 | 0.0 | 12830 | 16 | 0.1 | 18811 | 16 | 0.1 |
| Any hospital dialysis | 5981 | 15 | 0.3 | 12830 | 98 | 0.8 | 18811 | 113 | 0.6 |
| Any hospital bed admission | 5981 | 2682 | 44.8 | 12830 | 7311 | 57.0 | 18811 | 9993 | 53.1 |
| Elective bed admission without HF primary diagnosis | 5981 | 1314 | 22.0 | 12830 | 3567 | 27.8 | 18811 | 4881 | 25.9 |
| Emergency bed admission without HF primary diagnosis | 5981 | 1940 | 32.4 | 12830 | 5593 | 43.6 | 18811 | 7533 | 40.0 |
| Emergency non-HF bed admission (one day only) | 5981 | 137 | 2.3 | 12830 | 1045 | 8.1 | 18811 | 1182 | 6.3 |
| Emergency non-HF bed admission (at least one night) | 5981 | 1873 | 31.3 | 12830 | 5173 | 40.3 | 18811 | 7046 | 37.5 |
| A&E visit ending in admission* | 5981 | 0 | 0.0 | 12830 | 5023 | 39.2 | 18811 | 5023 | 26.7 |
| A&E visit not ending in admission* | 5981 | 0 | 0.0 | 12830 | 3051 | 23.8 | 18811 | 3051 | 16.2 |
| OPD appointment attended* | 5981 | 0 | 0.0 | 12830 | 9468 | 73.8 | 18811 | 9468 | 50.3 |
| OPD appointment not attended* | 5981 | 0 | 0.0 | 12830 | 4353 | 33.9 | 18811 | 4353 | 23.1 |
| OPD appointment for cardiology* | 5981 | 0 | 0.0 | 12830 | 3422 | 26.7 | 18811 | 3422 | 18.2 |
| OPD appointment specialty* | 5981 | 0 | 0.0 | 12830 | 9705 | 75.6 | 18811 | 9705 | 51.6 |
| 4+ minute GP appointment | 5981 | 4700 | 78.6 | 12830 | 12251 | 95.5 | 18811 | 16951 | 90.1 |
| 4+ minute practice nurse appointment | 5981 | 2614 | 43.7 | 12830 | 9243 | 72.0 | 18811 | 11857 | 63.0 |
| Home visit appointment | 5981 | 2108 | 35.2 | 12830 | 4317 | 33.6 | 18811 | 6425 | 34.2 |
| Out of hours appointment | 5981 | 862 | 14.4 | 12830 | 2378 | 18.5 | 18811 | 3240 | 17.2 |
| GP reported non-attendance | 5981 | 221 | 3.7 | 12830 | 560 | 4.4 | 18811 | 781 | 4.2 |
| Practice nurse reported non-attendance | 5981 | 110 | 1.8 | 12830 | 539 | 4.2 | 18811 | 649 | 3.5 |
| CPRD recorded A&E visit | 5981 | 348 | 5.8 | 12830 | 3970 | 30.9 | 18811 | 4318 | 23.0 |
| CPRD recorded OPD appointment | 5981 | 1504 | 25.1 | 12830 | 8228 | 64.1 | 18811 | 9732 | 51.7 |
| Heart failure review | 5981 | 0 | 0.0 | 12830 | 2 | 0.0 | 18811 | 2 | 0.0 |
| Beta blockers (BNF Chapter 2.4) | 5981 | 1453 | 24.3 | 12830 | 5013 | 39.1 | 18811 | 6466 | 34.4 |
| Thiazide-related diuretics (BNF Chapter 2.2.1) | 5981 | 1158 | 19.4 | 12830 | 2434 | 19.0 | 18811 | 3592 | 19.1 |
| Loop diuretics (BNF Chapter 2.2.2) | 5981 | 2349 | 39.3 | 12830 | 5504 | 42.9 | 18811 | 7853 | 41.7 |
| Aldosterone antagonists (spironolactone or eplerenone) | 5981 | 186 | 3.1 | 12830 | 709 | 5.5 | 18811 | 895 | 4.8 |
| Renin-angiotensin system (RAS) drugs (BNF Chapter 2.5.5) | 5981 | 1903 | 31.8 | 12830 | 6967 | 54.3 | 18811 | 8870 | 47.2 |
| Glucocorticoid therapy (BNF Chapter 6.3.2) | 5981 | 870 | 14.5 | 12830 | 2464 | 19.2 | 18811 | 3334 | 17.7 |
| Atypical antipsychotics (BNF Chapter 4.2.1.2 or drug names) | 5981 | 59 | 1.0 | 12830 | 146 | 1.1 | 18811 | 205 | 1.1 |
| **Primary CCS groups for HES admissions in previous year:** |  |  |  |  |  |  |  |  |  |
| 086 Cataract | 5981 | 176 | 2.9 | 12830 | 410 | 3.2 | 18811 | 586 | 3.1 |
| 122 Pneumonia (except that caused by tuberculosis or sexually transmitted diseases | 5981 | 70 | 1.2 | 12830 | 474 | 3.7 | 18811 | 544 | 2.9 |
| 127 Chronic obstructive pulmonary disease and bronchiectasis | 5981 | 122 | 2.0 | 12830 | 342 | 2.7 | 18811 | 464 | 2.5 |
| 134 Other upper respiratory disease | 5981 | 89 | 1.5 | 12830 | 345 | 2.7 | 18811 | 434 | 2.3 |

* Recorded from HES, so zero for the first window, before HES began to record OPD and A&E

Table A2. Harrell’s c-indices with respect to survival time for the 8 models in the combined test set of patients diagnosed in Window 2 (166 practices, 6796 patients) and their differences

| *Diagnosis setting and model or simple-complicated contrast* | *Harrell's c or difference* | *(95%* | *CI)* | *P* |
| --- | --- | --- | --- | --- |
| **Harrell’s c indices:** |  |  |  |  |
| Gompertz, Unseparated | 0.7039 | (0.6933, | 0.7146) | 3.4x10^-83^ |
| Gompertz, Separated by diagnosis setting | 0.7031 | (0.6918, | 0.7144) | 3.4x10^-79^ |
| Gompertz, Separated by diagnosis setting and time window | 0.7002 | (0.6887, | 0.7117) | 4.9x10^-77^ |
| Gompertz, Separated by diagnosis setting, Window 2 only | 0.7007 | (0.6891, | 0.7123) | 8.9x10^-77^ |
| Weibull, Unseparated | 0.7026 | (0.6920, | 0.7132) | 5.2x10^-83^ |
| Weibull, Separated by diagnosis setting | 0.7025 | (0.6915, | 0.7135) | 1.8x10^-80^ |
| Weibull, Separated by diagnosis setting and time window | 0.7011 | (0.6900, | 0.7123) | 1.5x10^-79^ |
| Weibull, Separated by diagnosis setting, Window 2 only | 0.7015 | (0.6902, | 0.7128) | 1.1x10^-78^ |
| Cox, Unseparated | 0.7029 | (0.6921, | 0.7137) | 4.5x10^-82^ |
| Cox, Separated by diagnosis setting | 0.7032 | (0.6919, | 0.7145) | 3.7x10^-79^ |
| Cox, Separated by diagnosis setting and time window | 0.7010 | (0.6896, | 0.7124) | 3.9x10^-78^ |
| Cox, Separated by diagnosis setting, Window 2 only | 0.7014 | (0.6899, | 0.7129) | 1.2x10^-77^ |

Table A2 shows that discrimination differed very little by hazard function. The next two plots show that calibration for the Cox model (see main manuscript) was better than that for the Weibull or Gompertz.

Figure A1.1 Decile plots of predicted and observed k-year survival probabilities in the test set from the unseparated Gompertz model


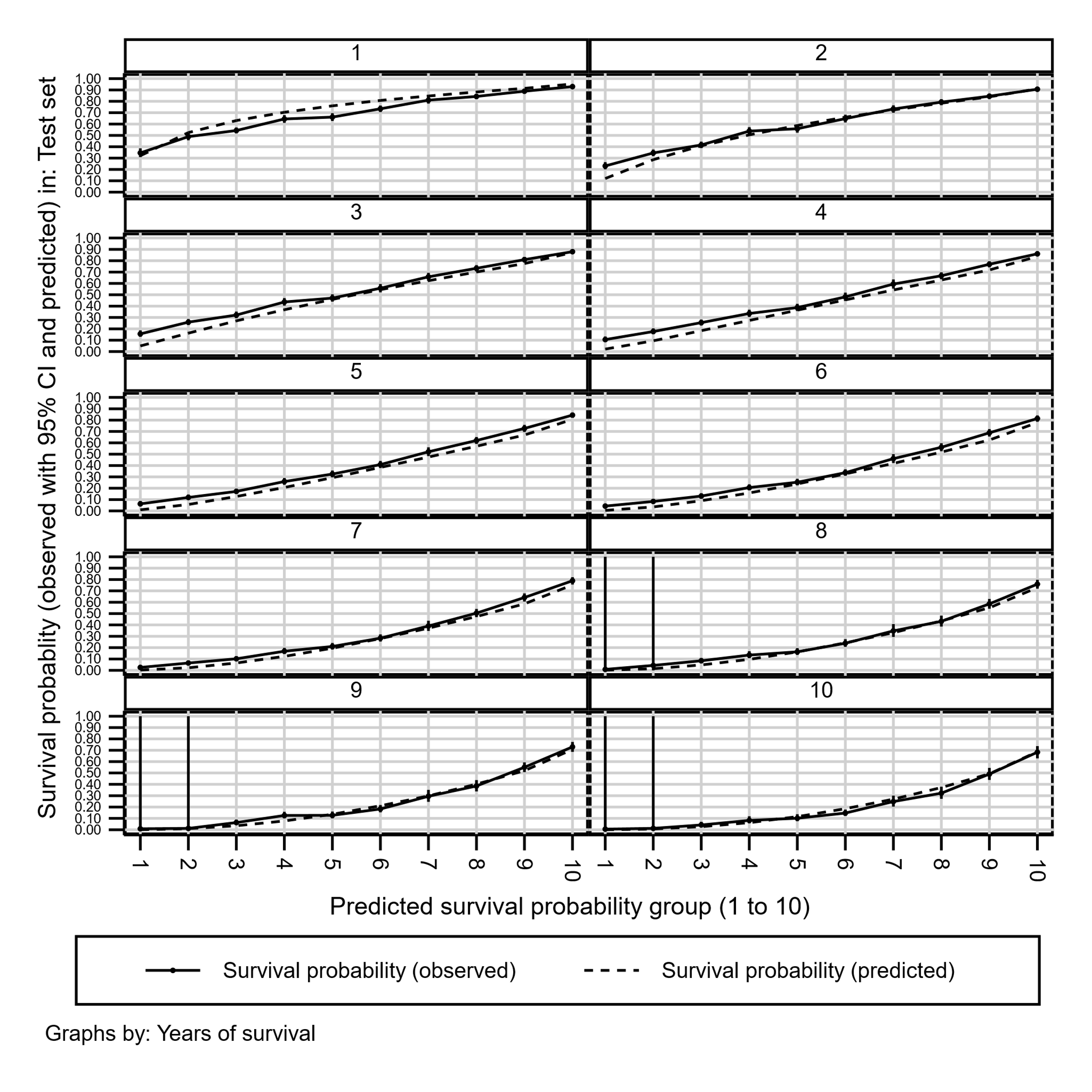


Tables A3.1 to A3.4 show all the hazard ratios for each window and diagnosis setting.

**Table A3.1. Regression of survival to all-cause death with respect to candidate model 3 (Cox model, HF patients diagnosed in primary care setting in Window 1) (82 practices, 1575 patients, 1410 deaths in training set)**

| *Predictor* | *Hazard ratio* | *(95%* | *CI)* | *P* |
| --- | --- | --- | --- | --- |
|  |  |  |  |  |
| **Attained age in diagnosis year:** |  |  |  |  |
| 50 | 0.194 | (0.043, | 0.880) | .034 |
| 60 | 0.537 | (0.327, | 0.880) | .014 |
| 70 (ref) | 1.000 | (ref) |  |  |
| 80 | 1.673 | (1.376, | 2.036) | 2.6x10^-7^ |
| 90 | 2.862 | (2.157, | 3.797) | 3.1x10^-13^ |
| **Female gender (by age):** |  |  |  |  |
| 50 | 2.155 | (0.924, | 5.026) | .076 |
| 60 | 0.956 | (0.625, | 1.462) | .83 |
| 70 | 0.604 | (0.492, | 0.742) | 1.5x10^-6^ |
| 80 | 0.753 | (0.666, | 0.852) | 6.6x10^-6^ |
| 90 | 0.919 | (0.770, | 1.097) | .35 |
| **BMI (age 50):** |  |  |  |  |
| 20 | 1.164 | (0.033, | 40.451) | .93 |
| 25 (ref) | 1.000 | (ref) |  |  |
| 30 | 1.160 | (0.312, | 4.320) | .82 |
| 35 | 0.780 | (0.158, | 3.847) | .76 |
| 40 | 0.592 | (0.081, | 4.356) | .61 |
| **BMI (age 60):** |  |  |  |  |
| 20 | 2.045 | (1.218, | 3.435) | .0068 |
| 25 (ref) | 1.000 | (ref) |  |  |
| 30 | 0.716 | (0.484, | 1.060) | .095 |
| 35 | 0.876 | (0.450, | 1.704) | .7 |
| 40 | 1.360 | (0.547, | 3.380) | .51 |
| **BMI (age 70):** |  |  |  |  |
| 20 | 1.647 | (1.223, | 2.217) | .001 |
| 25 (ref) | 1.000 | (ref) |  |  |
| 30 | 1.028 | (0.828, | 1.275) | .8 |
| 35 | 1.061 | (0.779, | 1.446) | .71 |
| 40 | 1.153 | (0.715, | 1.859) | .56 |
| **BMI (age 80):** |  |  |  |  |
| 20 | 1.076 | (0.832, | 1.391) | .58 |
| 25 (ref) | 1.000 | (ref) |  |  |
| 30 | 0.973 | (0.821, | 1.154) | .75 |
| 35 | 1.002 | (0.752, | 1.335) | .99 |
| 40 | 0.885 | (0.518, | 1.513) | .66 |
| **BMI (age 90):** |  |  |  |  |
| 20 | 1.110 | (0.802, | 1.536) | .53 |
| 25 (ref) | 1.000 | (ref) |  |  |
| 30 | 0.730 | (0.543, | 0.981) | .037 |
| 35 | 0.632 | (0.292, | 1.368) | .24 |
| 40 | 1.836 | (0.711, | 4.739) | .21 |
| **Systolic BP (age 50):** |  |  |  |  |
| 120 | 0.826 | (0.331, | 2.065) | .68 |
| 130 | 0.919 | (0.611, | 1.383) | .69 |
| 140 (ref) | 1.000 | (ref) |  |  |
| 150 | 0.956 | (0.499, | 1.830) | .89 |
| 160 | 0.704 | (0.152, | 3.269) | .65 |
| **Systolic BP (age 60):** |  |  |  |  |
| 120 | 1.133 | (0.661, | 1.943) | .65 |
| 130 | 1.048 | (0.814, | 1.350) | .72 |
| 140 (ref) | 1.000 | (ref) |  |  |
| 150 | 0.957 | (0.803, | 1.141) | .63 |
| 160 | 0.932 | (0.635, | 1.368) | .72 |
| **Systolic BP (age 70):** |  |  |  |  |
| 120 | 1.108 | (0.921, | 1.332) | .28 |
| 130 | 1.026 | (0.948, | 1.110) | .52 |
| 140 (ref) | 1.000 | (ref) |  |  |
| 150 | 1.000 | (0.944, | 1.059) | 1 |
| 160 | 1.018 | (0.894, | 1.158) | .79 |
| **Systolic BP (age 80):** |  |  |  |  |
| 120 | 1.090 | (0.913, | 1.301) | .34 |
| 130 | 1.048 | (0.957, | 1.148) | .31 |
| 140 (ref) | 1.000 | (ref) |  |  |
| 150 | 0.955 | (0.907, | 1.005) | .079 |
| 160 | 0.921 | (0.840, | 1.009) | .078 |
| **Systolic BP (age 90):** |  |  |  |  |
| 120 | 1.085 | (0.896, | 1.314) | .4 |
| 130 | 1.048 | (0.954, | 1.153) | .33 |
| 140 (ref) | 1.000 | (ref) |  |  |
| 150 | 0.992 | (0.929, | 1.060) | .82 |
| 160 | 1.018 | (0.893, | 1.161) | .79 |
| **Electronic frailty index (eFI):** |  |  |  |  |
| .12 | 0.934 | (0.823, | 1.059) | .29 |
| .24 (ref) | 1.000 | (ref) |  |  |
| .36 | 1.047 | (0.832, | 1.318) | .69 |
| .48 | 0.707 | (0.212, | 2.352) | .57 |
| **IMD 2010 twentile (imputed):** |  |  |  |  |
| 1 | 1.167 | (0.971, | 1.402) | .099 |
| 5 | 0.939 | (0.784, | 1.124) | .49 |
| 10 (ref) | 1.000 | (ref) |  |  |
| 15 | 0.998 | (0.868, | 1.149) | .98 |
| 20 | 1.075 | (0.811, | 1.426) | .62 |
| **Imputed QRISK-3 smoking category (imputed):** |  |  |  |  |
| non-smoker (ref) | 1.000 | (ref) |  |  |
| ex-smoker | 0.866 | (0.719, | 1.042) | .13 |
| light smoker (less than 10) | 1.126 | (0.694, | 1.829) | .63 |
| moderate smoker (10 to 19) | 0.952 | (0.835, | 1.084) | .46 |
| heavy smoker (20 or over) | 1.676 | (1.019, | 2.756) | .042 |
| **Diabetes status:** |  |  |  |  |
| No diabetes (ref) | 1.000 | (ref) |  |  |
| Type 1 diabetes | 1.215 | (0.958, | 1.542) | .11 |
| Type 2 diabetes | 1.735 | (1.438, | 2.093) | 8.6x10^-9^ |
| **HES ethnicity (white or non-white):** |  |  |  |  |
| White (ref) | 1.000 | (ref) |  |  |
| Non-white | 0.822 | (0.587, | 1.150) | .25 |
| Unknown | 2.685 | (2.257, | 3.195) | 8.5x10^-29^ |
| **Binary predictors:** |  |  |  |  |
| Comorbidity: 1 Atrial fibrillation | 0.921 | (0.801, | 1.059) | .25 |
| Comorbidity: 2 Arrhythmia other than atrial fibrillation | 0.922 | (0.752, | 1.132) | .44 |
| Comorbidity: 4 Hypertension | 0.908 | (0.816, | 1.011) | .077 |
| Comorbidity: 5 Renal diseases | 1.535 | (1.132, | 2.080) | .0058 |
| Comorbidity: 6 Myocarditis | 0.983 | (0.506, | 1.909) | .96 |
| Comorbidity: 7 Acute myocardial infarction | 0.924 | (0.766, | 1.113) | .4 |
| Comorbidity: 8 Congenital heart disease | 1.168 | (0.879, | 1.551) | .28 |
| Comorbidity: 9 Coronary heart disease | 0.919 | (0.805, | 1.049) | .21 |
| Comorbidity: 10 Chronic pulmonary disease | 1.187 | (0.999, | 1.409) | .051 |
| Comorbidity: 11 Stroke | 1.164 | (0.923, | 1.467) | .2 |
| Comorbidity: 12 Peripheral vascular disease | 1.281 | (1.056, | 1.554) | .012 |
| Living alone | 1.083 | (0.789, | 1.487) | .62 |
| Widowed or bereaved | 0.956 | (0.798, | 1.146) | .63 |
| Presence of: Breathlessness/SOB/SOBE | 0.908 | (0.796, | 1.035) | .15 |
| Presence of: Fatigue | 1.085 | (0.901, | 1.307) | .39 |
| Presence of: Ankle swelling | 0.951 | (0.805, | 1.123) | .55 |
| CABG | 0.613 | (0.218, | 1.723) | .35 |
| PTCA | 0.546 | (0.320, | 0.932) | .027 |
| Pacemaker | 0.680 | (0.393, | 1.175) | .17 |
| ICD (ref) | 1.000 | (ref) |  |  |
| Any hospital dialysis | 1.477 | (0.567, | 3.851) | .42 |
| Elective bed admission without HF primary diagnosis | 1.185 | (1.030, | 1.363) | .017 |
| Emergency non-HF bed admission (one day only) | 0.987 | (0.683, | 1.427) | .94 |
| Emergency non-HF bed admission (at least one night) | 1.257 | (1.067, | 1.480) | .0062 |
| 4+ minute GP appointment | 0.934 | (0.768, | 1.136) | .49 |
| 4+ minute practice nurse appointment | 0.886 | (0.764, | 1.027) | .11 |
| Home visit appointment | 1.301 | (1.151, | 1.469) | .000024 |
| Out of hours appointment | 1.121 | (0.947, | 1.327) | .18 |
| GP reported non-attendance | 0.808 | (0.544, | 1.198) | .29 |
| Practice nurse reported non-attendance | 1.319 | (0.912, | 1.908) | .14 |
| CPRD recorded A&E visit | 0.831 | (0.601, | 1.148) | .26 |
| CPRD recorded OPD appointment | 1.053 | (0.920, | 1.204) | .45 |
| Beta blockers (BNF Chapter 2.4) | 0.911 | (0.810, | 1.023) | .12 |
| Thiazide-related diuretics (BNF Chapter 2.2.1) | 1.093 | (0.955, | 1.252) | .2 |
| Loop diuretics (BNF Chapter 2.2.2) | 1.081 | (0.958, | 1.220) | .21 |
| Aldosterone antagonists (spironolactone or eplerenone) | 1.039 | (0.744, | 1.451) | .82 |
| Renin-angiotensin system (RAS) drugs (BNF Chapter 2.5.5) | 0.935 | (0.822, | 1.063) | .31 |
| Glucocorticoid therapy (BNF Chapter 6.3.2) | 1.048 | (0.878, | 1.251) | .6 |
| Atypical antipsychotics (BNF Chapter 4.2.1.2 or drug names) | 1.580 | (1.091, | 2.287) | .015 |
| CCS group: 086 Cataract | 0.966 | (0.673, | 1.386) | .85 |
| CCS group: 122 Pneumonia (except that caused by TB or STD) | 1.450 | (0.916, | 2.293) | .11 |
| CCS group: 127 COPD and bronchiectasis | 1.204 | (0.641, | 2.264) | .56 |
| CCS group: 134 Other upper respiratory disease | 1.044 | (0.705, | 1.547) | .83 |

**Table A3.2. Regression of survival to all-cause death with respect to candidate model 5 (Cox model, HF patients diagnosed in primary care setting in Window 2) (158 practices, 1775 patients, 875 deaths in training set)**

| *Predictor* | *Hazard ratio* | *(95%* | *CI)* | *P* |
| --- | --- | --- | --- | --- |
| **Attained age in diagnosis year:** |  |  |  |  |
| 50 | 0.165 | (0.019, | 1.416) | .1 |
| 60 | 0.828 | (0.512, | 1.340) | .44 |
| 70 (ref) | 1.000 | (ref) |  |  |
| 80 | 2.652 | (1.994, | 3.526) | 2.0x10^-11^ |
| 90 | 8.279 | (5.044, | 13.591) | 6.3x10^-17^ |
| **Female gender (by age):** |  |  |  |  |
| 50 | 3.179 | (1.012, | 9.992) | .048 |
| 60 | 0.929 | (0.567, | 1.524) | .77 |
| 70 | 0.738 | (0.547, | 0.995) | .046 |
| 80 | 0.680 | (0.544, | 0.850) | .0007 |
| 90 | 0.705 | (0.539, | 0.921) | .011 |
| **BMI (age 50):** |  |  |  |  |
| 20 | 0.333 | (0.048, | 2.334) | .27 |
| 25 (ref) | 1.000 | (ref) |  |  |
| 30 | 1.478 | (0.445, | 4.914) | .52 |
| 35 | 1.383 | (0.345, | 5.545) | .65 |
| 40 | 0.921 | (0.187, | 4.549) | .92 |
| **BMI (age 60):** |  |  |  |  |
| 20 | 2.374 | (1.576, | 3.577) | .000036 |
| 25 (ref) | 1.000 | (ref) |  |  |
| 30 | 1.020 | (0.690, | 1.508) | .92 |
| 35 | 1.431 | (0.840, | 2.437) | .19 |
| 40 | 1.972 | (1.008, | 3.858) | .047 |
| **BMI (age 70):** |  |  |  |  |
| 20 | 2.112 | (1.594, | 2.798) | 1.9x10^-7^ |
| 25 (ref) | 1.000 | (ref) |  |  |
| 30 | 1.163 | (0.877, | 1.542) | .29 |
| 35 | 1.319 | (0.886, | 1.963) | .17 |
| 40 | 1.419 | (0.885, | 2.273) | .15 |
| **BMI (age 80):** |  |  |  |  |
| 20 | 1.315 | (1.082, | 1.597) | .0058 |
| 25 (ref) | 1.000 | (ref) |  |  |
| 30 | 0.859 | (0.701, | 1.053) | .14 |
| 35 | 0.845 | (0.621, | 1.149) | .28 |
| 40 | 0.779 | (0.506, | 1.198) | .26 |
| **BMI (age 90):** |  |  |  |  |
| 20 | 1.476 | (1.154, | 1.889) | .0019 |
| 25 (ref) | 1.000 | (ref) |  |  |
| 30 | 0.601 | (0.477, | 0.756) | .000014 |
| 35 | 0.579 | (0.368, | 0.913) | .019 |
| 40 | 0.713 | (0.407, | 1.250) | .24 |
| **Systolic BP (age 50):** |  |  |  |  |
| 120 | 1.299 | (0.416, | 4.059) | .65 |
| 130 | 0.871 | (0.450, | 1.685) | .68 |
| 140 (ref) | 1.000 | (ref) |  |  |
| 150 | 2.381 | (0.810, | 7.003) | .11 |
| 160 | 8.500 | (1.057, | 68.350) | .044 |
| **Systolic BP (age 60):** |  |  |  |  |
| 120 | 1.138 | (0.782, | 1.656) | .5 |
| 130 | 1.005 | (0.841, | 1.202) | .95 |
| 140 (ref) | 1.000 | (ref) |  |  |
| 150 | 1.147 | (0.832, | 1.581) | .4 |
| 160 | 1.179 | (0.628, | 2.212) | .61 |
| **Systolic BP (age 70):** |  |  |  |  |
| 120 | 1.620 | (1.185, | 2.214) | .0025 |
| 130 | 1.221 | (1.054, | 1.414) | .0078 |
| 140 (ref) | 1.000 | (ref) |  |  |
| 150 | 1.022 | (0.815, | 1.281) | .85 |
| 160 | 1.158 | (0.721, | 1.861) | .54 |
| **Systolic BP (age 80):** |  |  |  |  |
| 120 | 1.206 | (1.018, | 1.428) | .03 |
| 130 | 1.067 | (0.985, | 1.155) | .11 |
| 140 (ref) | 1.000 | (ref) |  |  |
| 150 | 1.016 | (0.883, | 1.170) | .83 |
| 160 | 1.100 | (0.811, | 1.492) | .54 |
| **Systolic BP (age 90):** |  |  |  |  |
| 120 | 0.741 | (0.579, | 0.950) | .018 |
| 130 | 0.864 | (0.776, | 0.961) | .0074 |
| 140 (ref) | 1.000 | (ref) |  |  |
| 150 | 0.996 | (0.843, | 1.177) | .96 |
| 160 | 0.877 | (0.596, | 1.290) | .51 |
| **Electronic frailty index (eFI):** |  |  |  |  |
| .12 | 0.843 | (0.669, | 1.063) | .15 |
| .24 (ref) | 1.000 | (ref) |  |  |
| .36 | 1.281 | (1.083, | 1.514) | .0038 |
| .48 | 1.468 | (0.728, | 2.958) | .28 |
| **IMD 2010 twentile (imputed):** |  |  |  |  |
| 1 | 0.763 | (0.568, | 1.026) | .074 |
| 5 | 1.043 | (0.850, | 1.280) | .69 |
| 10 (ref) | 1.000 | (ref) |  |  |
| 15 | 1.135 | (0.932, | 1.383) | .21 |
| 20 | 1.091 | (0.820, | 1.450) | .55 |
| **Imputed QRISK-3 smoking category (imputed):** |  |  |  |  |
| non-smoker (ref) | 1.000 | (ref) |  |  |
| ex-smoker | 1.169 | (0.995, | 1.374) | .057 |
| light smoker (less than 10) | 1.370 | (0.845, | 2.222) | .2 |
| moderate smoker (10 to 19) | 1.442 | (1.165, | 1.786) | .00079 |
| heavy smoker (20 or over) | 2.066 | (1.205, | 3.542) | .0084 |
| **Diabetes status:** |  |  |  |  |
| No diabetes (ref) | 1.000 | (ref) |  |  |
| Type 1 diabetes | 1.323 | (0.942, | 1.858) | .11 |
| Type 2 diabetes | 1.021 | (0.814, | 1.282) | .86 |
| **HES ethnicity (white or non-white):** |  |  |  |  |
| White (ref) | 1.000 | (ref) |  |  |
| Non-white | 1.025 | (0.607, | 1.731) | .93 |
| Unknown | 1.994 | (1.043, | 3.812) | .037 |
| **Binary predictors:** |  |  |  |  |
| Comorbidity: 1 Atrial fibrillation | 0.950 | (0.808, | 1.117) | .53 |
| Comorbidity: 2 Arrhythmia other than atrial fibrillation | 0.985 | (0.823, | 1.179) | .87 |
| Comorbidity: 4 Hypertension | 1.135 | (0.961, | 1.342) | .14 |
| Comorbidity: 5 Renal diseases | 1.049 | (0.900, | 1.222) | .54 |
| Comorbidity: 6 Myocarditis | 0.707 | (0.395, | 1.264) | .24 |
| Comorbidity: 7 Acute myocardial infarction | 1.101 | (0.867, | 1.399) | .43 |
| Comorbidity: 8 Congenital heart disease | 0.798 | (0.330, | 1.929) | .62 |
| Comorbidity: 9 Coronary heart disease | 0.853 | (0.703, | 1.035) | .11 |
| Comorbidity: 10 Chronic pulmonary disease | 1.242 | (1.014, | 1.521) | .036 |
| Comorbidity: 11 Stroke | 0.938 | (0.745, | 1.182) | .59 |
| Comorbidity: 12 Peripheral vascular disease | 1.571 | (1.255, | 1.966) | .000079 |
| Living alone | 0.982 | (0.736, | 1.311) | .9 |
| Widowed or bereaved | 0.833 | (0.620, | 1.117) | .22 |
| Presence of: Breathlessness/SOB/SOBE | 0.879 | (0.736, | 1.050) | .16 |
| Presence of: Fatigue | 0.682 | (0.561, | 0.829) | .00012 |
| Presence of: Ankle swelling | 1.217 | (1.003, | 1.476) | .047 |
| CABG | 0.926 | (0.388, | 2.213) | .86 |
| PTCA | 0.523 | (0.319, | 0.857) | .01 |
| Pacemaker | 0.755 | (0.475, | 1.200) | .23 |
| ICD | 0.000 | (0.000, | 0.000) | 0 |
| Elective bed admission without HF primary diagnosis | 1.162 | (0.987, | 1.368) | .071 |
| Emergency non-HF bed admission (one day only) | 0.980 | (0.724, | 1.328) | .9 |
| Emergency non-HF bed admission (at least one night) | 1.047 | (0.875, | 1.255) | .61 |
| 4+ minute GP appointment | 1.460 | (0.878, | 2.426) | .14 |
| 4+ minute practice nurse appointment | 1.054 | (0.877, | 1.266) | .58 |
| Home visit appointment | 1.557 | (1.310, | 1.852) | 5.1x10^-7^ |
| Out of hours appointment | 0.970 | (0.810, | 1.160) | .74 |
| GP reported non-attendance | 1.010 | (0.707, | 1.443) | .96 |
| Practice nurse reported non-attendance | 0.937 | (0.622, | 1.413) | .76 |
| CPRD recorded A&E visit | 1.067 | (0.884, | 1.289) | .5 |
| CPRD recorded OPD appointment | 0.921 | (0.775, | 1.094) | .35 |
| Beta blockers (BNF Chapter 2.4) | 0.922 | (0.776, | 1.095) | .35 |
| Thiazide-related diuretics (BNF Chapter 2.2.1) | 0.877 | (0.717, | 1.073) | .2 |
| Loop diuretics (BNF Chapter 2.2.2) | 1.063 | (0.903, | 1.253) | .46 |
| Aldosterone antagonists (spironolactone or eplerenone) | 1.856 | (1.382, | 2.493) | .00004 |
| Renin-angiotensin system (RAS) drugs (BNF Chapter 2.5.5) | 0.872 | (0.733, | 1.038) | .12 |
| Glucocorticoid therapy (BNF Chapter 6.3.2) | 1.022 | (0.851, | 1.228) | .82 |
| Atypical antipsychotics (BNF Chapter 4.2.1.2 or drug names) | 1.709 | (0.606, | 4.823) | .31 |
| CCS group: 086 Cataract | 1.165 | (0.782, | 1.734) | .45 |
| CCS group: 122 Pneumonia (except that caused by TB or STD) | 1.079 | (0.635, | 1.833) | .78 |
| CCS group: 127 COPD and bronchiectasis | 2.227 | (1.363, | 3.638) | .0014 |
| CCS group: 134 Other upper respiratory disease | 0.868 | (0.522, | 1.441) | .58 |

**Table A3.3. Regression of survival to all-cause death with respect to candidate model 7 (Cox model, HF patients diagnosed in hospital setting in Window 1) (84 practices, 1617 patients, 1459 deaths in training set)**

| *Predictor* | *Hazard ratio* | *(95%* | *CI)* | *P* |
| --- | --- | --- | --- | --- |
| **Attained age in diagnosis year:** |  |  |  |  |
| 50 | 0.026 | (0.006, | 0.105) | 3.1x10^-7^ |
| 60 | 0.412 | (0.273, | 0.623) | .000026 |
| 70 (ref) | 1.000 | (ref) |  |  |
| 80 | 1.995 | (1.577, | 2.523) | 8.5x10^-9^ |
| 90 | 3.138 | (2.209, | 4.458) | 1.7x10^-10^ |
| **Female gender (by age):** |  |  |  |  |
| 50 | 0.655 | (0.332, | 1.293) | .22 |
| 60 | 0.648 | (0.456, | 0.921) | .016 |
| 70 | 0.833 | (0.662, | 1.047) | .12 |
| 80 | 0.811 | (0.687, | 0.958) | .014 |
| 90 | 0.848 | (0.677, | 1.062) | .15 |
| **BMI (age 50):** |  |  |  |  |
| 20 | 12.876 | (2.452, | 67.596) | .0025 |
| 25 (ref) | 1.000 | (ref) |  |  |
| 30 | 5.764 | (1.768, | 18.788) | .0037 |
| 35 | 18.963 | (4.000, | 89.905) | .00021 |
| 40 | 39.111 | (8.640, | 177.050) | 1.9x10^-6^ |
| **BMI (age 60):** |  |  |  |  |
| 20 | 1.511 | (0.868, | 2.632) | .14 |
| 25 (ref) | 1.000 | (ref) |  |  |
| 30 | 0.997 | (0.632, | 1.574) | .99 |
| 35 | 1.878 | (1.076, | 3.279) | .027 |
| 40 | 4.055 | (2.272, | 7.238) | 2.2x10^-6^ |
| **BMI (age 70):** |  |  |  |  |
| 20 | 1.232 | (0.831, | 1.826) | .3 |
| 25 (ref) | 1.000 | (ref) |  |  |
| 30 | 1.035 | (0.835, | 1.282) | .75 |
| 35 | 0.968 | (0.680, | 1.376) | .85 |
| 40 | 0.842 | (0.492, | 1.441) | .53 |
| **BMI (age 80):** |  |  |  |  |
| 20 | 1.543 | (1.288, | 1.850) | 2.6x10^-6^ |
| 25 (ref) | 1.000 | (ref) |  |  |
| 30 | 0.990 | (0.838, | 1.169) | .91 |
| 35 | 0.960 | (0.724, | 1.273) | .78 |
| 40 | 0.956 | (0.664, | 1.378) | .81 |
| **BMI (age 90):** |  |  |  |  |
| 20 | 1.319 | (1.040, | 1.674) | .023 |
| 25 (ref) | 1.000 | (ref) |  |  |
| 30 | 0.884 | (0.684, | 1.143) | .35 |
| 35 | 0.661 | (0.339, | 1.289) | .22 |
| 40 | 0.900 | (0.291, | 2.780) | .85 |
| **Systolic BP (age 50):** |  |  |  |  |
| 120 | 2.121 | (1.151, | 3.908) | .016 |
| 130 | 1.400 | (0.985, | 1.990) | .06 |
| 140 (ref) | 1.000 | (ref) |  |  |
| 150 | 0.878 | (0.666, | 1.157) | .35 |
| 160 | 0.921 | (0.552, | 1.534) | .75 |
| **Systolic BP (age 60):** |  |  |  |  |
| 120 | 1.567 | (1.123, | 2.187) | .0082 |
| 130 | 1.248 | (1.021, | 1.525) | .031 |
| 140 (ref) | 1.000 | (ref) |  |  |
| 150 | 0.990 | (0.843, | 1.162) | .9 |
| 160 | 1.151 | (0.837, | 1.582) | .39 |
| **Systolic BP (age 70):** |  |  |  |  |
| 120 | 1.063 | (0.834, | 1.354) | .62 |
| 130 | 1.022 | (0.910, | 1.147) | .71 |
| 140 (ref) | 1.000 | (ref) |  |  |
| 150 | 0.987 | (0.922, | 1.058) | .72 |
| 160 | 0.980 | (0.872, | 1.101) | .73 |
| **Systolic BP (age 80):** |  |  |  |  |
| 120 | 0.797 | (0.680, | 0.935) | .0053 |
| 130 | 0.911 | (0.845, | 0.982) | .015 |
| 140 (ref) | 1.000 | (ref) |  |  |
| 150 | 0.991 | (0.943, | 1.041) | .71 |
| 160 | 0.910 | (0.829, | 0.999) | .047 |
| **Systolic BP (age 90):** |  |  |  |  |
| 120 | 1.110 | (0.855, | 1.441) | .43 |
| 130 | 1.043 | (0.918, | 1.184) | .52 |
| 140 (ref) | 1.000 | (ref) |  |  |
| 150 | 0.970 | (0.898, | 1.047) | .43 |
| 160 | 0.940 | (0.821, | 1.076) | .37 |
| **Electronic frailty index (eFI):** |  |  |  |  |
| .12 | 0.998 | (0.882, | 1.129) | .97 |
| .24 (ref) | 1.000 | (ref) |  |  |
| .36 | 1.284 | (1.107, | 1.491) | .00099 |
| .48 | 2.804 | (1.422, | 5.526) | .0029 |
| **IMD 2010 twentile (imputed):** |  |  |  |  |
| 1 | 1.140 | (0.892, | 1.457) | .29 |
| 5 | 1.006 | (0.852, | 1.188) | .95 |
| 10 (ref) | 1.000 | (ref) |  |  |
| 15 | 1.219 | (1.039, | 1.432) | .015 |
| 20 | 1.442 | (1.106, | 1.880) | .0069 |
| **Imputed QRISK-3 smoking category (imputed):** |  |  |  |  |
| non-smoker (ref) | 1.000 | (ref) |  |  |
| ex-smoker | 0.893 | (0.713, | 1.117) | .32 |
| light smoker (less than 10) | 1.043 | (0.649, | 1.677) | .86 |
| moderate smoker (10 to 19) | 1.029 | (0.906, | 1.169) | .66 |
| heavy smoker (20 or over) | 1.695 | (1.299, | 2.213) | .0001 |
| **Diabetes status:** |  |  |  |  |
| No diabetes (ref) | 1.000 | (ref) |  |  |
| Type 1 diabetes | 1.170 | (0.915, | 1.495) | .21 |
| Type 2 diabetes | 1.263 | (1.042, | 1.530) | .017 |
| **HES ethnicity (white or non-white):** |  |  |  |  |
| White (ref) | 1.000 | (ref) |  |  |
| Non-white | 0.810 | (0.529, | 1.241) | .33 |
| Unknown | 2.441 | (2.059, | 2.894) | 9.7x10^-25^ |
| **Binary predictors:** |  |  |  |  |
| Comorbidity: 1 Atrial fibrillation | 0.901 | (0.803, | 1.011) | .077 |
| Comorbidity: 2 Arrhythmia other than atrial fibrillation | 0.841 | (0.724, | 0.978) | .024 |
| Comorbidity: 4 Hypertension | 0.956 | (0.860, | 1.063) | .41 |
| Comorbidity: 5 Renal diseases | 1.395 | (1.136, | 1.712) | .0015 |
| Comorbidity: 6 Myocarditis | 0.712 | (0.389, | 1.303) | .27 |
| Comorbidity: 7 Acute myocardial infarction | 0.917 | (0.767, | 1.097) | .35 |
| Comorbidity: 8 Congenital heart disease | 0.523 | (0.233, | 1.176) | .12 |
| Comorbidity: 9 Coronary heart disease | 0.914 | (0.805, | 1.039) | .17 |
| Comorbidity: 10 Chronic pulmonary disease | 1.312 | (1.129, | 1.524) | .00038 |
| Comorbidity: 11 Stroke | 1.047 | (0.831, | 1.318) | .7 |
| Comorbidity: 12 Peripheral vascular disease | 1.701 | (1.447, | 1.999) | 1.1x10^-10^ |
| Living alone | 0.986 | (0.803, | 1.211) | .89 |
| Widowed or bereaved | 1.017 | (0.763, | 1.355) | .91 |
| Presence of: Breathlessness/SOB/SOBE | 0.910 | (0.789, | 1.049) | .19 |
| Presence of: Fatigue | 0.859 | (0.700, | 1.054) | .14 |
| Presence of: Ankle swelling | 1.142 | (0.948, | 1.376) | .16 |
| CABG | 0.642 | (0.254, | 1.623) | .35 |
| PTCA | 0.504 | (0.165, | 1.541) | .23 |
| Pacemaker | 1.575 | (0.943, | 2.630) | .083 |
| ICD (ref) | 1.000 | (ref) |  |  |
| Any hospital dialysis | 1.284 | (0.328, | 5.029) | .72 |
| Elective bed admission without HF primary diagnosis | 1.096 | (0.967, | 1.242) | .15 |
| Emergency non-HF bed admission (one day only) | 1.649 | (1.109, | 2.452) | .013 |
| Emergency non-HF bed admission (at least one night) | 1.077 | (0.947, | 1.226) | .26 |
| 4+ minute GP appointment | 0.920 | (0.786, | 1.077) | .3 |
| 4+ minute practice nurse appointment | 1.025 | (0.916, | 1.147) | .67 |
| Home visit appointment | 1.285 | (1.149, | 1.438) | .000012 |
| Out of hours appointment | 1.029 | (0.886, | 1.196) | .7 |
| GP reported non-attendance | 1.013 | (0.715, | 1.436) | .94 |
| Practice nurse reported non-attendance | 0.952 | (0.623, | 1.453) | .82 |
| CPRD recorded A&E visit | 0.960 | (0.805, | 1.144) | .65 |
| CPRD recorded OPD appointment | 0.944 | (0.835, | 1.067) | .35 |
| Beta blockers (BNF Chapter 2.4) | 0.951 | (0.843, | 1.072) | .41 |
| Thiazide-related diuretics (BNF Chapter 2.2.1) | 1.119 | (0.975, | 1.283) | .11 |
| Loop diuretics (BNF Chapter 2.2.2) | 1.086 | (0.950, | 1.242) | .23 |
| Aldosterone antagonists (spironolactone or eplerenone) | 0.932 | (0.718, | 1.211) | .6 |
| Renin-angiotensin system (RAS) drugs (BNF Chapter 2.5.5) | 0.985 | (0.885, | 1.096) | .78 |
| Glucocorticoid therapy (BNF Chapter 6.3.2) | 1.101 | (0.952, | 1.272) | .2 |
| Atypical antipsychotics (BNF Chapter 4.2.1.2 or drug names) | 1.416 | (0.975, | 2.056) | .068 |
| CCS group: 086 Cataract | 1.003 | (0.716, | 1.405) | .99 |
| CCS group: 122 Pneumonia (except that caused by TB or STD) | 1.184 | (0.764, | 1.835) | .45 |
| CCS group: 127 COPD and bronchiectasis | 1.195 | (0.882, | 1.619) | .25 |
| CCS group: 134 Other upper respiratory disease | 0.798 | (0.512, | 1.245) | .32 |

**Table A3.4. Regression of survival to all-cause death with respect to candidate model 9 (Cox model, HF patients diagnosed in hospital setting in Window 2) (164 practices, 4256 patients, 3083 deaths in training set)**

| *Predictor* | *Hazard ratio* | *(95%* | *CI)* | *P* |
| --- | --- | --- | --- | --- |
| **Attained age in diagnosis year:** |  |  |  |  |
| 50 | 0.487 | (0.300, | 0.791) | .0036 |
| 60 | 0.591 | (0.472, | 0.741) | 4.8x10^-6^ |
| 70 (ref) | 1.000 | (ref) |  |  |
| 80 | 1.772 | (1.551, | 2.024) | 3.6x10^-17^ |
| 90 | 2.945 | (2.360, | 3.675) | 1.1x10^-21^ |
| **Female gender (by age):** |  |  |  |  |
| 50 | 1.638 | (1.033, | 2.599) | .036 |
| 60 | 1.331 | (1.040, | 1.703) | .023 |
| 70 | 0.923 | (0.782, | 1.089) | .34 |
| 80 | 0.779 | (0.704, | 0.863) | 1.5x10^-6^ |
| 90 | 0.757 | (0.681, | 0.841) | 2.3x10^-7^ |
| **BMI (age 50):** |  |  |  |  |
| 20 | 1.925 | (1.323, | 2.802) | .00063 |
| 25 (ref) | 1.000 | (ref) |  |  |
| 30 | 0.695 | (0.493, | 0.979) | .038 |
| 35 | 0.694 | (0.416, | 1.156) | .16 |
| 40 | 0.809 | (0.442, | 1.478) | .49 |
| **BMI (age 60):** |  |  |  |  |
| 20 | 1.931 | (1.573, | 2.371) | 3.4x10^-10^ |
| 25 (ref) | 1.000 | (ref) |  |  |
| 30 | 0.812 | (0.685, | 0.963) | .016 |
| 35 | 0.828 | (0.640, | 1.071) | .15 |
| 40 | 0.894 | (0.645, | 1.238) | .5 |
| **BMI (age 70):** |  |  |  |  |
| 20 | 1.623 | (1.394, | 1.889) | 4.1x10^-10^ |
| 25 (ref) | 1.000 | (ref) |  |  |
| 30 | 0.819 | (0.729, | 0.919) | .00072 |
| 35 | 0.744 | (0.621, | 0.890) | .0013 |
| 40 | 0.726 | (0.567, | 0.930) | .011 |
| **BMI (age 80):** |  |  |  |  |
| 20 | 1.397 | (1.278, | 1.528) | 2.3x10^-13^ |
| 25 (ref) | 1.000 | (ref) |  |  |
| 30 | 0.833 | (0.761, | 0.912) | .00008 |
| 35 | 0.799 | (0.699, | 0.915) | .0011 |
| 40 | 0.841 | (0.686, | 1.031) | .096 |
| **BMI (age 90):** |  |  |  |  |
| 20 | 1.171 | (1.047, | 1.309) | .0055 |
| 25 (ref) | 1.000 | (ref) |  |  |
| 30 | 0.858 | (0.774, | 0.951) | .0034 |
| 35 | 0.728 | (0.608, | 0.872) | .00056 |
| 40 | 0.654 | (0.493, | 0.867) | .0032 |
| **Systolic BP (age 50):** |  |  |  |  |
| 120 | 1.305 | (0.901, | 1.889) | .16 |
| 130 | 1.122 | (0.932, | 1.351) | .22 |
| 140 (ref) | 1.000 | (ref) |  |  |
| 150 | 1.038 | (0.808, | 1.332) | .77 |
| 160 | 1.202 | (0.653, | 2.213) | .56 |
| **Systolic BP (age 60):** |  |  |  |  |
| 120 | 1.049 | (0.860, | 1.280) | .64 |
| 130 | 1.032 | (0.934, | 1.140) | .54 |
| 140 (ref) | 1.000 | (ref) |  |  |
| 150 | 0.942 | (0.835, | 1.063) | .33 |
| 160 | 0.879 | (0.653, | 1.184) | .4 |
| **Systolic BP (age 70):** |  |  |  |  |
| 120 | 0.997 | (0.882, | 1.126) | .96 |
| 130 | 1.004 | (0.949, | 1.061) | .9 |
| 140 (ref) | 1.000 | (ref) |  |  |
| 150 | 0.946 | (0.891, | 1.004) | .067 |
| 160 | 0.860 | (0.747, | 0.991) | .037 |
| **Systolic BP (age 80):** |  |  |  |  |
| 120 | 1.067 | (0.979, | 1.164) | .14 |
| 130 | 1.036 | (0.994, | 1.081) | .095 |
| 140 (ref) | 1.000 | (ref) |  |  |
| 150 | 0.949 | (0.905, | 0.996) | .033 |
| 160 | 0.895 | (0.805, | 0.995) | .04 |
| **Systolic BP (age 90):** |  |  |  |  |
| 120 | 1.152 | (1.046, | 1.268) | .004 |
| 130 | 1.072 | (1.025, | 1.122) | .0026 |
| 140 (ref) | 1.000 | (ref) |  |  |
| 150 | 0.943 | (0.890, | 0.999) | .046 |
| 160 | 0.908 | (0.800, | 1.032) | .14 |
| **Electronic frailty index (eFI):** |  |  |  |  |
| .12 | 0.918 | (0.838, | 1.007) | .069 |
| .24 (ref) | 1.000 | (ref) |  |  |
| .36 | 0.898 | (0.823, | 0.980) | .016 |
| .48 | 0.804 | (0.628, | 1.029) | .083 |
| **IMD 2010 twentile (imputed):** |  |  |  |  |
| 1 | 1.022 | (0.876, | 1.192) | .78 |
| 5 | 1.059 | (0.952, | 1.178) | .29 |
| 10 (ref) | 1.000 | (ref) |  |  |
| 15 | 1.056 | (0.948, | 1.176) | .33 |
| 20 | 1.105 | (0.946, | 1.292) | .21 |
| **Imputed QRISK-3 smoking category (imputed):** |  |  |  |  |
| non-smoker (ref) | 1.000 | (ref) |  |  |
| ex-smoker | 1.015 | (0.939, | 1.098) | .71 |
| light smoker (less than 10) | 1.103 | (0.850, | 1.431) | .46 |
| moderate smoker (10 to 19) | 1.200 | (1.071, | 1.344) | .0017 |
| heavy smoker (20 or over) | 1.594 | (1.222, | 2.080) | .00059 |
| **Diabetes status:** |  |  |  |  |
| No diabetes (ref) | 1.000 | (ref) |  |  |
| Type 1 diabetes | 1.322 | (1.131, | 1.544) | .00045 |
| Type 2 diabetes | 1.221 | (1.108, | 1.347) | .000062 |
| **HES ethnicity (white or non-white):** |  |  |  |  |
| White (ref) | 1.000 | (ref) |  |  |
| Non-white | 0.614 | (0.479, | 0.786) | .00011 |
| Unknown | 2.344 | (1.703, | 3.226) | 1.7x10^-7^ |
| **Binary predictors:** |  |  |  |  |
| Comorbidity: 1 Atrial fibrillation | 1.020 | (0.956, | 1.089) | .55 |
| Comorbidity: 2 Arrhythmia other than atrial fibrillation | 0.872 | (0.795, | 0.956) | .0036 |
| Comorbidity: 4 Hypertension | 1.073 | (0.961, | 1.199) | .21 |
| Comorbidity: 5 Renal diseases | 1.298 | (1.213, | 1.389) | 4.8x10^-14^ |
| Comorbidity: 6 Myocarditis | 0.813 | (0.600, | 1.102) | .18 |
| Comorbidity: 7 Acute myocardial infarction | 1.000 | (0.910, | 1.098) | .99 |
| Comorbidity: 8 Congenital heart disease | 0.673 | (0.368, | 1.232) | .2 |
| Comorbidity: 9 Coronary heart disease | 0.848 | (0.775, | 0.929) | .00037 |
| Comorbidity: 10 Chronic pulmonary disease | 1.212 | (1.101, | 1.333) | .000087 |
| Comorbidity: 11 Stroke | 0.931 | (0.817, | 1.062) | .29 |
| Comorbidity: 12 Peripheral vascular disease | 1.120 | (0.999, | 1.255) | .051 |
| Living alone | 0.953 | (0.851, | 1.067) | .4 |
| Widowed or bereaved | 0.952 | (0.821, | 1.103) | .51 |
| Presence of: Breathlessness/SOB/SOBE | 0.943 | (0.867, | 1.025) | .17 |
| Presence of: Fatigue | 0.970 | (0.861, | 1.094) | .62 |
| Presence of: Ankle swelling | 1.013 | (0.918, | 1.118) | .8 |
| CABG | 0.462 | (0.253, | 0.842) | .012 |
| PTCA | 0.663 | (0.463, | 0.948) | .024 |
| Pacemaker | 1.020 | (0.802, | 1.298) | .87 |
| ICD | 1.015 | (0.297, | 3.467) | .98 |
| Any hospital dialysis | 1.472 | (0.880, | 2.464) | .14 |
| Elective bed admission without HF primary diagnosis | 1.049 | (0.946, | 1.163) | .36 |
| Emergency non-HF bed admission (one day only) | 1.072 | (0.942, | 1.221) | .29 |
| Emergency non-HF bed admission (at least one night) | 1.291 | (1.180, | 1.413) | 3.0x10^-8^ |
| 4+ minute GP appointment | 1.057 | (0.821, | 1.360) | .67 |
| 4+ minute practice nurse appointment | 0.879 | (0.810, | 0.955) | .0023 |
| Home visit appointment | 1.394 | (1.279, | 1.519) | 3.6x10^-14^ |
| Out of hours appointment | 0.999 | (0.907, | 1.099) | .98 |
| GP reported non-attendance | 1.067 | (0.874, | 1.304) | .52 |
| Practice nurse reported non-attendance | 0.931 | (0.776, | 1.117) | .44 |
| CPRD recorded A&E visit | 0.965 | (0.889, | 1.047) | .39 |
| CPRD recorded OPD appointment | 0.945 | (0.871, | 1.027) | .18 |
| Beta blockers (BNF Chapter 2.4) | 0.915 | (0.838, | 0.999) | .049 |
| Thiazide-related diuretics (BNF Chapter 2.2.1) | 1.001 | (0.914, | 1.096) | .99 |
| Loop diuretics (BNF Chapter 2.2.2) | 1.070 | (0.993, | 1.154) | .077 |
| Aldosterone antagonists (spironolactone or eplerenone) | 1.220 | (1.042, | 1.428) | .013 |
| Renin-angiotensin system (RAS) drugs (BNF Chapter 2.5.5) | 0.886 | (0.819, | 0.959) | .0027 |
| Glucocorticoid therapy (BNF Chapter 6.3.2) | 1.099 | (0.992, | 1.219) | .072 |
| Atypical antipsychotics (BNF Chapter 4.2.1.2 or drug names) | 1.087 | (0.756, | 1.562) | .65 |
| CCS group: 086 Cataract | 1.071 | (0.850, | 1.349) | .56 |
| CCS group: 122 Pneumonia (except that caused by TB or STD) | 1.249 | (0.985, | 1.582) | .066 |
| CCS group: 127 COPD and bronchiectasis | 1.008 | (0.820, | 1.238) | .94 |
| CCS group: 134 Other upper respiratory disease | 0.925 | (0.763, | 1.122) | .43 |

Figure A2.2 Decile plots of predicted and observed k-year survival probabilities in the test set from the unseparated Weibull model.


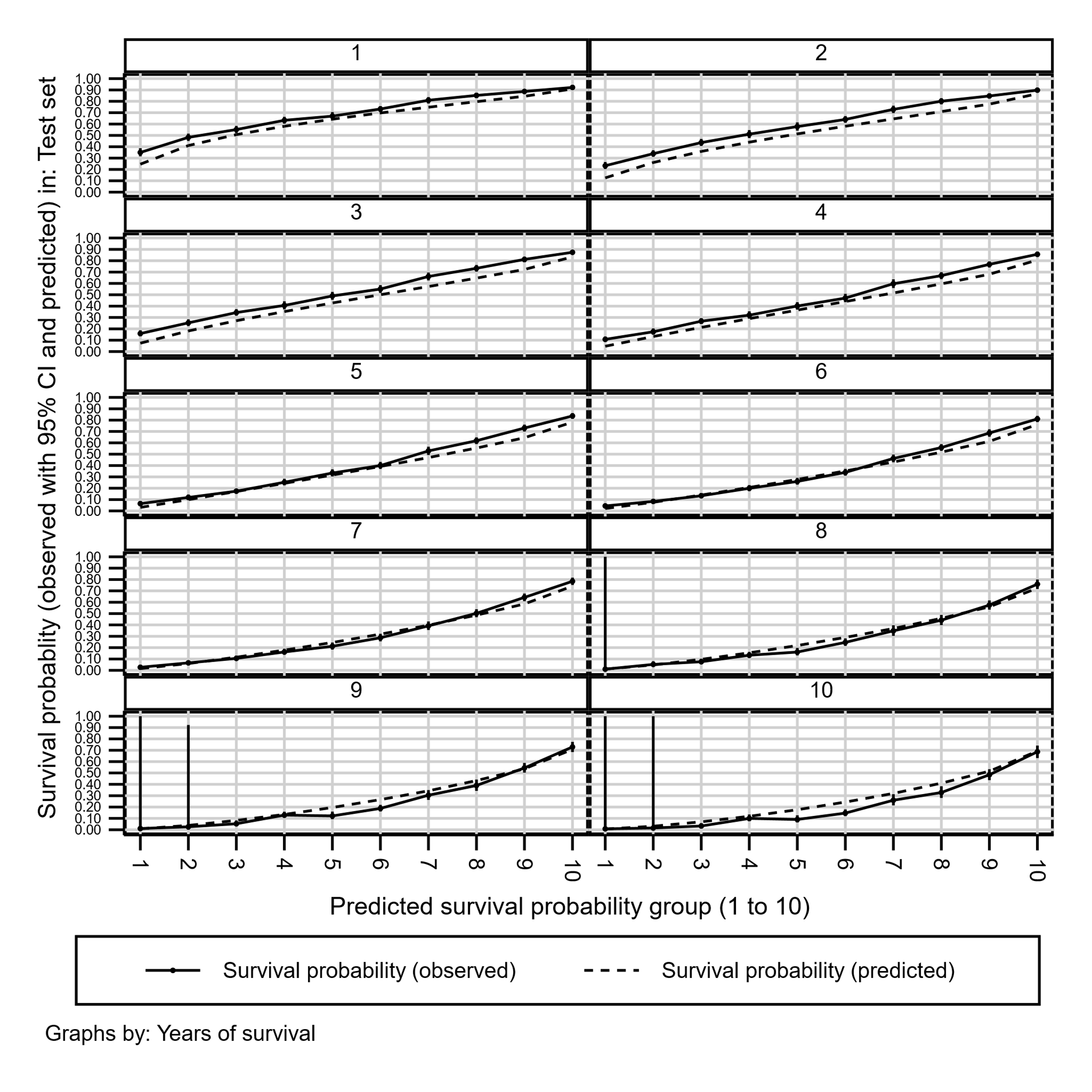

Supplement: Supplementary file 1 — Appendix S1. Supporting Information. [file EHF2-10-824-s001.docx]
